# Phenotypic, genomic, and transcriptional characterization of *Streptococcus pneumoniae* interacting with human pharyngeal cells

**DOI:** 10.1186/1471-2164-14-383

**Published:** 2013-06-09

**Authors:** Sheila Z Kimaro Mlacha, Sandra Romero-Steiner, Julie C Dunning Hotopp, Nikhil Kumar, Nadeeza Ishmael, David R Riley, Umar Farooq, Todd H Creasy, Luke J Tallon, Xinyue Liu, Cynthia S Goldsmith, Jacquelyn Sampson, George M Carlone, Susan K Hollingshead, J Anthony G Scott, Hervé Tettelin

**Affiliations:** 1 Kenya Medical Research Institute, Wellcome Trust Research Programme, Kilifi, Kenya; 2 Department of Microbiology and Immunology, Institute for Genome Sciences, University of Maryland School of Medicine, 801 W. Baltimore Street, Baltimore, MD 21201, USA; 3 Division of Bacterial Diseases, Centers for Disease Control and Prevention, Atlanta, GA, USA; 4 Division of High-Consequence Pathogens and Pathology, Centers for Disease Control and Prevention, Atlanta, GA, USA; 5 Department of Microbiology, University of Alabama at Birmingham, Birmingham, AL, USA; 6 Nuffield Department of Clinical Medicine, University of Oxford, Oxford, UK; 7 Present address: Respiratory & Meningeal Pathogens Research Unit, University of the Witwatersrand/Medical Research Council, Johannesburg, South Africa

**Keywords:** *Streptococcus pneumoniae*, Gene expression, Microarray, Adherence, Invasion, Genome, Mutagenesis, SP_1922, *Ply* operon

## Abstract

**Background:**

*Streptococcus pneumoniae* is a leading cause of childhood morbidity and mortality worldwide, despite the availability of effective pneumococcal vaccines. Understanding the molecular interactions between the bacterium and the host will contribute to the control and prevention of pneumococcal disease.

**Results:**

We used a combination of adherence assays, mutagenesis and functional genomics to identify novel factors involved in adherence. By contrasting these processes in two pneumococcal strains, TIGR4 and G54, we showed that adherence and invasion capacities vary markedly by strain. Electron microscopy showed more adherent bacteria in association with membranous pseudopodia in the TIGR4 strain. Operons for cell wall phosphorylcholine incorporation (*lic),* manganese transport (*psa*) and phosphate utilization (*phn*) were up-regulated in both strains on exposure to epithelial cells. Pneumolysin, pili, stress protection genes (*adhC*-*czcD*) and genes of the type II fatty acid synthesis pathway were highly expressed in the naturally more invasive strain, TIGR4. Deletion mutagenesis of five gene regions identified as regulated in this study revealed attenuation in adherence. Most strikingly, ∆SP_1922 which was predicted to contain a B-cell epitope and revealed significant attenuation in adherence, appeared to be expressed as a part of an operon that includes the gene encoding the cytoplasmic pore-forming toxin and vaccine candidate, pneumolysin.

**Conclusion:**

This work identifies a list of novel potential pneumococcal adherence determinants.

## Background

*Streptococcus pneumoniae* (the pneumococcus) is a leading cause of community-acquired pneumonia, meningitis, sepsis and otitis media. It colonizes the human nasopharynx asymptomatically, but can spread to the middle ear and lungs, or penetrate the epithelial cells and enter the bloodstream leading to invasive disease. Worldwide *S. pneumoniae* is responsible for >14.5 M cases of invasive disease annually and up to 11% of all deaths in children [1,2].

Adherence of the pneumococcus to the nasopharyngeal epithelium is essential to successful colonization and constitutes the first step in the invasive route of this pathogen. Previously, pneumococcal adherence and invasion has been studied by gene expression analyses using RT-PCR [3] or microarrays [4-7]. Despite the identification of several key adherence factors, including pneumococcal surface adhesin A (PsaA), pneumococcal serine repeat protein (PsrP), choline binding proteins (CbpA, CbpE), pilus protein (RrgA), plasminogen- and fibronectin-binding proteins (PfbA and PfbB), pneumococcal adherence and virulence factor A and B (PavA and PavB) [8-15], our understanding of the molecular mechanisms of pneumococcal adherence and invasion remains incomplete. Our research aims to improve our understanding of these mechanisms.

We measured variation in the adherence of two pneumococcal strains (TIGR4 and G54) to an epithelial cell line from the human pharynx (Detroit 562). We also examined the pathogen-host interaction in each of these two strains with electron microscopy. Next, we studied the genome-wide transcriptional response of *S. pneumoniae* as it adheres to and invades the D562 cells *in vitro.* We did this by comparing transcription profiles of pneumococcal strains exposed to either D562 cells or simple broth, and by comparing transcription profiles of pneumococci that successfully adhered to D562 cells to those that did not. These studies support the role of known adherence determinants and identified potential novel determinants, some of which have no predicted function. Using deletion mutagenesis, we also demonstrate the possible functional relevance of five of these genes.

## Methods

### Pneumococcal strains

TIGR4, a serotype 4 clinical isolate taken from blood, and G54, a serotype 19F isolate from human sputum, were routinely grown as previously described [8]. Colonies were visualized in a stereoscope with transmitted light as previously described [16] and only strains that were transparent in phenotype were used in this study. Viable counts were performed to determine the exact number of CFU per micro-titer well to be used in each experiment.

### *In vitro* adherence assay

Detroit 562 (D562) cells, human pharyngeal carcinoma epithelial cells, were purchased from the American Type Culture Collection (CCL-138) and grown and maintained as previously described [8]. Adherence of *S. pneumoniae* to D562 cells was assessed as previously described [8]. Briefly, D562 cells (10^5^/ml) were seeded in 96-well tissue culture treated plates (200 μl per well) and grown to confluence. The final cell yield was 1.2 x 10^5^ cells/well after 6 days of incubation resulting in a multiplicity of infection (MOI) of 0.01. Monolayers were washed once with 125 μl/well of minimal essential medium with Eagle’s salts (EMEM) without L-glutamine and supplemented with 7% fetal bovine serum (Atlas Biologicals, Fort Collins, CO). To the washed monolayer of each well, 80 μl of EMEM was added, followed by 20 μl/well of bacterial suspension (10^3^ bacteria/well). Plates were incubated for 2 h at 37°C in a 5% CO_2_ incubator to allow for adherence then washed 5 X with phosphate-buffered saline (PBS) with 0.2% bovine serum albumin (BSA) to remove non-adherent pneumococci. A 65 μl volume of THYE (Todd-Hewitt broth supplemented with 0.5% yeast extract), 0.8% agar and 0.1% 2,3,5-triphenyl tetrazolium chloride (TTC; Difco Laboratories), was added and the plates were incubated overnight at 37°C in a 5% CO_2_ incubator. The number of colonies of *S. pneumoniae* adhering to D562 cells was counted using an automated colony counter (AlphaImager; Alpha Innotech, CA) and adherence was expressed as 100 X (CFU of adherent bacteria / CFU of inoculated bacteria) per well. These counts are inclusive of bacteria that were internalized within the cells. Values for 4 replicate wells were averaged and 3 independent experiments were performed. Differences in adherence capacity between TIGR4 and G54 were tested by Student’s *t*-test.

For microarray experiments, aliquots (1 ml) of pneumococcal TIGR4 and G54 strains containing 10^7^ bacteria were centrifuged, washed in PBS, and re-suspended in 15 ml of EMEM. Confluent D562 cells, grown in 175 cm^2^ cell culture flasks (Corning Costar Co., MA, USA), were inoculated with these strains and incubated for 2 h at 37°C in a 5% CO_2_ incubator. Non-adherent bacteria (contained in the spent cell culture medium) were removed and collected by differential centrifugation to remove eukaryotic cellular debris. Epithelial cells were washed 3X with PBS and then treated immediately with 10 ml of RNAprotect (Qiagen, Germany). For experiments with G54, adherent bacteria were dissociated from host cells by treatment with trypsin-EDTA; for TIGR4, which adhered and invaded to D562 cells extremely efficiently, dissociation was achieved by lysis with 0.1% (w/v) saponin in PBS followed by sonication using 5 s pulses for 1 min. Bacteria were subsequently harvested by differential centrifugation (1st step: 800 x g for 5 min to remove mammalian cells, 2nd step: 4400 rpm for 10 min). Control bacteria, which were not exposed to host cells, were suspended in EMEM medium and then prepared in parallel and treated identically to adherent and non-adherent bacteria. They were also treated with 10 ml of RNAprotect to minimize RNA metabolism and degradation. Pellets were stored at −80°C.

The difference in treatments between G54 and TIGR4 stems from the chronology of the experiments. We began by testing the adherence and expression of the G54 strain. Then we attempted to apply the same method to recover the adherent population of the TIGR4 strain, but we were unable to retrieve enough TIGR4 bacteria for extraction of sufficient amounts of RNA. A significant proportion of bacteria remained lodged in the pharyngeal cells (as later evidenced by the electron microscopy results). Liberation of TIGR4 pneumococci therefore required selective lysis of eukaryotic cells by saponin treatment and sonication. For various reasons, we did not go back and repeat all G54 experiments, however it should be noted that prior to any detachment treatment all bacteria were incubated with 10 ml of RNAprotect (Qiagen) for immediate stabilization of RNA and to minimize RNA degradation. This incubation should prevent any change in bacterial RNA profiles during further treatments.

### Invasion assay

Internalization of bacteria into D562 cells was assessed by a gentamicin assay as previously described [17]. Briefly, confluent D562 cells were inoculated with *S. pneumoniae* as described in the adherence assay. After 1 h and 45 min incubation, the cells were treated with 200 μg/ml of gentamicin for 15 min to kill extracellular bacteria. Monolayers were washed 5 times with PBS with 0.2% BSA to remove non-adherent pneumococci and any residual gentamicin. As in the adherence assay, monolayers were overlaid with THYE agar with 1% TTC and incubated overnight at 37°C, 5% CO_2_. Internalized bacteria (CFU) were quantified using an automated colony counter (AlphaImager; Alpha Innotech, CA). Differences in invasion capacity between TIGR4 and G54 were tested by Student’s *t*-test.

Pneumococcal strains TIGR4 and G54 strains were tested for gentamicin sensitivity using gentamicin discs of 10 μg (Oxoid Ltd., Hampshire, England). Both strains had comparable gentamicin inhibition zone diameters of 10.3 mm for TIGR4 and 10 mm for G54. The time and concentration of antibiotics used in these experiments were optimized for the D562 cell line. Preliminary time-course experiments by Rajam *et al.*[17] (data not shown) showed that a 200 μg/ml concentration of gentamicin was sufficient to kill extracellular pneumococci in 15 minutes. Longer time-points resulted in gentamicin penetrating the Detroit 562 cells, which resulted in internal killing rather than extracellular killing.

### Electron microscopy

D562 cells, grown to confluence in 175 cm^2^ cell culture flasks (Corning Costar Co., MA), were inoculated as described above with 10^7^ bacteria (MOI of 10). After a 2-h incubation step, monolayers were washed with EM buffer (100 mM sodium phosphate, pH 7.6, 3 mM KCL, 3 mM MgCl_2_) and gently layered with 2.5% buffered glutaraldehyde (3 ml). The cells were scraped with a cell scraper (Corning Inc., Corning, NY) within 1 min and the cell suspension was centrifuged at 800 x g for 10 min at 24°C. The cell pellets were then incubated for 1 h at 4°C to fix them. Pellets were washed once with EM buffer and stored at 4°C. D562 cell specimens were embedded in a mixture of Eponate 12 and Araldite 502 [18] and thin sections of plastic-embedded cells were examined by electron microscopy.

### RNA preparation

Total RNA was isolated from three populations of RNA stabilized bacteria: adherent, non-adherent and bacteria growing freely in EMEM. Total RNA was isolated using TRIzol (Invitrogen Life Technologies, USA) in a lysing matrix containing silica beads and a FastPrep Instrument (Qbiogene, Inc, CA). Purification was done using i) phase separation with TRIzol and ii) column exclusion with the RNeasy Mini Kit (Qiagen, Germany) according to the manufacturers instructions. Contaminating genomic DNA was removed by DNase treatment. RNA concentration was determined using the NanoDrop ND-1000 spectrophotometer (NanoDrop Technologies, USA) and the integrity was assessed using the prokaryote and eukaryote total RNA chips on the Agilent 2100 Bioanalyzer (Agilent Technologies, Germany). Good quality RNAs were amplified using Genisphere’s SenseAMP kit (Genisphere Inc., PA) to increase the yield of RNA for microarray experiments.

### Probe preparation and microarray hybridization

The *S. pneumoniae* microarrays used in this study consisted of 3482 70-mer oligonucleotide probes from the genomes of 3 pneumococcal strains (TIGR4, R6 and G54) as well as 10 amplicons and 500 oligonucleotides (70-mers) from *Arabidopsis thaliana* which served as negative controls. All probes were aligned against the HG19 version of the human genome using BlastN and no significant hits were obtained. Probes were printed 5X on aminosilane-coated slides (SCHOTT Nexterion). The microarrays (version 6) were kindly provided by the Pathogen Functional Genomics Resource Center (PFGRC) at the J. Craig Venter Institute and experiments were performed as previously described [19]. Briefly, 2 μg of the total RNAs to be compared were reverse transcribed into single-stranded cDNA using 200 U Superscript II reverse transcriptase (Invitrogen), 6 μg random hexamers (Invitrogen), 1X first strand buffer (Invitrogen), 10 mM dithiothreitol (DTT), 0.5 mM dATP, 0.5 mM dCTP, 0.5 mM dGTP, 0.3 mM dTTP and 0.2 mM of aminoallyl-modified nucleotide (Invitrogen). The mixture was incubated overnight at 42°C and the reaction stopped by addition of 10 μl 0.5 M EDTA and 1 M NaOH. Amine-modified cDNA was purified using QIAquick PCR purification kit (Qiagen, Germany) followed by chemical labeling with Cy3- or Cy5-NHS-ester fluorescent dyes (Amersham-Pharmacia, Piscataway, NJ) in a final step. Slides were pre-hybridized in a 50 ml solution of 5X SSC, 0.1% SDS and 1% BSA for 30 min at 42°C, washed 4X in water and once in isopropanol, then dried by brief centrifugation. Labeled probes were re-suspended in hybridization buffer (30% formamide, 5X SSC, 0.1% SDS, 0.6 μg/μL salmon sperm DNA) and hybridized to the microarray slides in a 42°C water bath for 16–20 h. Slides were washed twice in a low stringency buffer (2X SSC, 0.1% SDS) at 55°C for 5 min, twice in a medium stringency buffer (0.1X SSC, 0.1% SDS) at room temperature for 5 min and finally twice in a high stringency buffer (0.1X SSC) at room temperature for 5 min, and then dried by brief centrifugation. Synthesized cDNA from each RNA sample from three independent cell cultures was hybridized on three separate microarray slides (biological replicates), and independently synthesized cDNA from each of these same RNA samples was hybridized in a repeat dye-swap experiment (technical replicates) to test technical reproducibility. Additional file 1 summarizes the experimental design.

### Data collection, normalization, and analysis

Dried slides were scanned using a GenePix 4000B dual-color laser scanner (Axon Instruments, CA, USA) and saved as two independent 16-bit TIFF files corresponding to the two labeled probes (Cy3 and Cy5). Data were analyzed using the TM4 microarray software suite [20]. Spot intensities were quantified using Spotfinder v3.1.1. The two channels were normalized using the iterative log mean centering algorithm implemented in the MIDAS software (v2.19) and the fluorescence ratios were calculated from the normalized values. Genes with significant changes in expression were identified using the significance analysis of microarrays (SAM) test implemented in the MeV software (v4.2) with ∆ = 1.18 for TIGR4 and ∆ = 1.98 for G54 (∆ cutoff corresponded to a false positive rate of 0%). Only genes that had ≥ 15 individual high quality data points were included in the analysis. A cutoff of mean fold change of ≥ 2 was used. This gene list was supplemented with genes that did not meet the fold threshold value but appeared to be co-regulated as a part of an operon. Up and down-regulated genes were classified into meaningful functional categories using EASE (MeV). EASE statistically calculates the probability that a biological theme (TIGR4 or G54 role categories) is over-represented in the gene list of interest compared to the representation of that theme on the microarray. In our study, *p-*values of <0.001 were considered significant (EASE, Fisher’s exact test with Bonferroni step down correction). Significance was determined for each strain separately.

SAM two-class paired analysis (analogous to a between subjects *t*-test) was used to identify genes exhibiting statistically significant differences in gene expression between adherent/medium-treated control and non-adherent/medium-treated control. A ratio of these gene expression ratios (referred to as ratio index) was calculated for these significantly regulated genes and an arbitrary threshold of 1.5 fold change was set on the ratio index.

### Validation of microarray data using real-time qRT-PCR

Reverse transcription was carried out using the QuantiTect Reverse Transcription Kit (Qiagen, Germany) in accordance with the manufacturer’s instructions. Briefly, 1 μg of total RNA was incubated in genomic DNA (gDNA) Wipeout Buffer (7X) and RNase-free water and incubated at 42°C for 2 min to remove contaminating gDNA. The cDNA was synthesized from the RNA using Quantiscript reverse transcriptase, Quantiscript RT buffer (5X) and a primer mix consisting of long random primers and oligo-dT. The reaction was incubated at 42°C for 15 min and then at 95°C for 3 min to inactivate the Quantiscript RT. Quantitative real-time PCR was performed as previously described [19]. Dilutions of the cDNA (0.25 μl of stock cDNA per 20 μl reaction) were used as template in a reaction containing 2X QuantiTect SYBR Green mix (Qiagen, Germany), RNase free water and gene-specific primers (Additional file 2). The qRT-PCR was conducted using an ABI7900HT machine (Applied Biosystems). The reactions were denatured at 95°C for 15 min followed by amplification with 45 cycles of 94°C for 15 s, 55°C for 30 s and 72°C for 30 s. The qRT-PCR data was analyzed using a comparative cycle threshold (ΔCt) method [21]. The average Ct values of a test sample were compared to that of a control sample (e.g. Ct_TIGR4 control_ – Ct_TIGR4 adherent_). The ΔCt was normalized to genes that did not exhibit any significant change in expression as identified by the microarray experiments: an average of SP_0002 (DNA polymerase), SP_0085 (ribosomal protein) and SP_2135 (ribosomal protein). Each sample of each biological replicate was tested three times.

### RNA Sequencing

The transcriptome of *S. pneumoniae* TIGR4 adhering to D562 host cells was determined using RNA-Seq on the Illumina Genome Analyzer platform as per the manufacturer’s instructions. Briefly, 10 μg of total RNA was partially depleted of rRNA using the MicrobeExpress kit (Ambion) then fragmented in a buffer with divalent cations at 94°C for 5 min. Double-stranded cDNA was synthesized from the fragmented RNA using SuperScript II (Invitrogen) and random primers. The fragment ends were repaired using T4 DNA polymerase and the DNA polymerase Klenow fragment in the presence dNTPs to remove 3′ overhangs and fill in 5′ overhangs. Subsequently, a 3′ “A” overhang was added using a DNA polymerase Klenow fragment lacking 3′ to 5′ exonuclease activity in the presence of dATP. Illumina adaptors with a single 3′ “T” overhang were ligated to both ends of the DNA fragments. cDNA of the appropriate size range was gel purified and amplified, and Illumina sequencing conducted with unpaired reads 36 nt in length. 13,404,147 high quality sequencing reads were obtained and 95% of them mapped to the reference *S. pneumoniae* TIGR4 complete genome sequence using the ELAND alignment algorithm (Illumina). The reads and alignment data were deposited at the NCBI Gene Expression Omnibus (GEO) database under accession number: GE44947.

### Construction of knockout mutants

In selecting genes for knockout experiments, the features we sought were one or more of the following: (i) significant up-regulation on contact with epithelial cells in the microarray experiments; (ii) up-regulation in the more adherent strain (TIGR4); (iii) ratio index (adherent/control) / (non-adherent/control) ≥ 1.5 in both strains; (iv) conservation among the sequenced strains of pneumococci with the exception of known regions of diversity; (v) no prior existing experimental evidence of association with pneumococcal adhesion; (vi) large hypothetical proteins; and (vii) plausibility of knocking out a gene that was part of an operon without causing a polar effect. Preparation of mutagenic constructs, restriction digestion, ligation and transformation were performed using the PCR ligation mutagenesis strategy (insertion deletion mutagenesis) previously described [22]. Briefly, two pairs of gene-specific primers, J1/J2 and J3/J4, were used to amplify the upstream and downstream flanking regions of a number of target genes. J2 and J3 were modified to contain KpnI and SacI restriction sites. A third pair of primers, KanF and rpslR, were used to amplify a 1.3-kb positive/negative selection cassette *kan-rpsL*^*+*^ that was previously introduced in *S. pneumoniae* strain 403 J [23]. The *kan-rpsL*^*+*^ cassette was comprised of a kanamycin resistant marker (*kan*) and a counter-selectable marker *rpsL*^*+*^ which conferred dominant streptomycin sensitivity in a streptomycin resistant pneumococcal strain. All the primers used in this study are listed in Additional file 3. Amplification proceeded for 34 cycles as follows: denaturation at 95°C for 30 s, annealing at 62°C (or 55°C depending on the primers) for 30 s, and elongation at 72°C for 1 min. Amplicons were purified, digested overnight at 37°C using KpnI or SacI restriction enzymes and purified again to completely inactivate the enzyme. The 3 digested PCR products were then ligated using 1 μg of T4 DNA ligase (Invitrogen, CA, USA) in ligase buffer at room temperature for 2 h.

### Transformation

Genetic transformation of the ligation products into *S. pneumoniae* strain TIGR4-Z5 (transparent phenotype of a TIGR4 strain) was performed as follows. A series of transformation stocks were prepared by growing *S. pneumoniae* in competence media (THYE with 0.2% BSA, 0.2% glucose, and 0.02% CaCl_2_) at 37°C in a 5% CO_2_ incubator to an OD_600nm_ of 0.4, and freezing down the cultures in 20% glycerol. A 1 ml frozen aliquot of transformation stocks was thawed out and grown in competence media to exponential phase (OD_600nm_ = 0.4) at 37°C in a 5% CO_2_ incubator. The cells were subsequently diluted in competence medium (1:100) and transformed with 10 μl of the ligation products and 500 ng of the competence peptide CSP-2 and incubated for 2 h at 37°C. Cells were then plated on blood agar plates with antibiotics and incubated for up to 48 h. *S. pneumoniae* strain Ali 9.2 containing a *kan-rpsL*^*+*^ cassette in place of the *ali* gene, was used as the positive control and a no-DNA control plate was included to check for background resistance activities in the recipient cells. Transformants were selected based on their resistance to kanamycin (Kn^R^; positive selection) and susceptibility to streptomycin (Sm^S^; negative selection). Confirmation of gene replacement was performed using colony PCR. Individual colonies that were Kn^R^ and Sm^S^ were used as template in a PCR reaction using two pairs of primers (J1/kanR and rpslF/J4). Presence of a PCR product of the expected size was indicative of the presence of the cassette in the mutant strain and at the correct location. Colonies with the correct size were re-grown in THYE broth and a glycerol stock was kept at −80°C for use in adherence assays. The adherence assay to test the ability of mutant strains to bind to D562 cells was similar to that described above.

### Hemolytic activity of TIGR4 wild type and ∆SP1922 isogenic mutant

*S. pneumoniae* TIGR4 wild type and ∆SP_1922 were grown to an OD_600nm_ of 0.4 in THYE. The cells were centrifuged at 3000 g for 20 min, and the cell pellets were washed once with PBS and re-suspended in 2 ml of PBS. Bacteria were sonicated for 5 min at 50% duty cycle using an ultrasonicator (Branson Sonifier 450, USA) in an ice container. Cell lysates were centrifuged at 20,000 × g for 30 min at 4°C and the supernatants (cell extract) were transferred to a clean tube. Serial dilutions of the cell extracts in PBS (1 ml volume) were prepared. Human erythrocytes were washed twice in cold PBS and were added to the cell extracts to yield a final concentration of 2% (v/v). The suspension was incubated at 37°C for 30 min then centrifuged gently at 800 g for 10 min. Supernatants were transferred to a clean microtiter plate and the absorbance of released hemoglobin was determined at 540 nm using a microplate spetrophotometer (Labsystems Multiskan RC, Fisher Scientific, MA). The positive control was saponin, a known hemolytic agent, and the negative control was 0.5% dimethyl sulfoxide (DMSO). The relative hemolytic activity (% lysis) at each concentration was determined using a control containing 2% (v/v) erythrocytes and deionized water that was considered as 100% hemolysis. All experiments were carried out in triplicate.

### RT-PCR confirmation of the SP_1922 - *ply* operon

Total RNA was isolated from *S. pneumoniae* strain TIGR4 grown in THYE using the Qiagen RNeasy Mini Kit (Qiagen, Germany) and treated with DNase on column and in solution. Reverse transcription was performed using SuperScript II Reverse Transcriptase (Invitrogen, Carlsbad, CA) following the manufacturer’s instructions. Total RNA (800 ng) was incubated with each of 10 gene-specific primers (Additional file 4) at 65°C for 5 min and quick chilled on ice. To this mixture was added 5X First-Strand Buffer and 0.1 M DTT, and the samples were incubated at 42°C for 2 min. SuperScript II RT was then added and the samples were incubated at 42°C for 50 min, followed by enzyme inactivation by heating to 70°C for 15 min. The 10 cDNAs were pooled and PCR was performed with NEB Taq 2X Master Mix (NEB, Ipswich, MA), following the standard protocol and using combinations of gene-specific primers spanning various regions within the pneumolysin operon (Additional file 4).

## Results

### Pneumococcal adhesion to and internalization within D562 pharyngeal cells

Adhesion of *S. pneumoniae* to D562 pharyngeal epithelial cell monolayers was assayed *in vitro,* and invasion was assessed based on gentamicin selection for intracellular bacteria. Two pneumococcal strains were tested, TIGR4 and G54. The mean adherence capacity to D562 cells was significantly higher (*p* value <0.05) for TIGR4 (545 CFU/well ± 40) than G54 (89 CFU/well ± 13). This translated to 54.5% of the inoculum for TIGR4 and 8.9% of the inoculum for G54. The internalization capacity also varied significantly (*p* value <0.05) between the 2 strains; 233 CFU/well ± 12 for TIGR4 (23.3% of the inoculum) and 3 CFU/well for G54 (0.3% of the inoculum). When we analyzed the growth patterns of TIGR4 and G54 during the 2-h assay, we found that some growth occurred in EMEM but the differences were not statistically significant up to 2 h (Additional file 5). We adjusted the adherence and invasion calculations for growth, resulting in values of 37.2% and 4.3% for TIGR4 and G54, respectively. Similarly, invasion was 15.8% and 0.14% for TIGR4 and G54, respectively.

**Figure 1:**
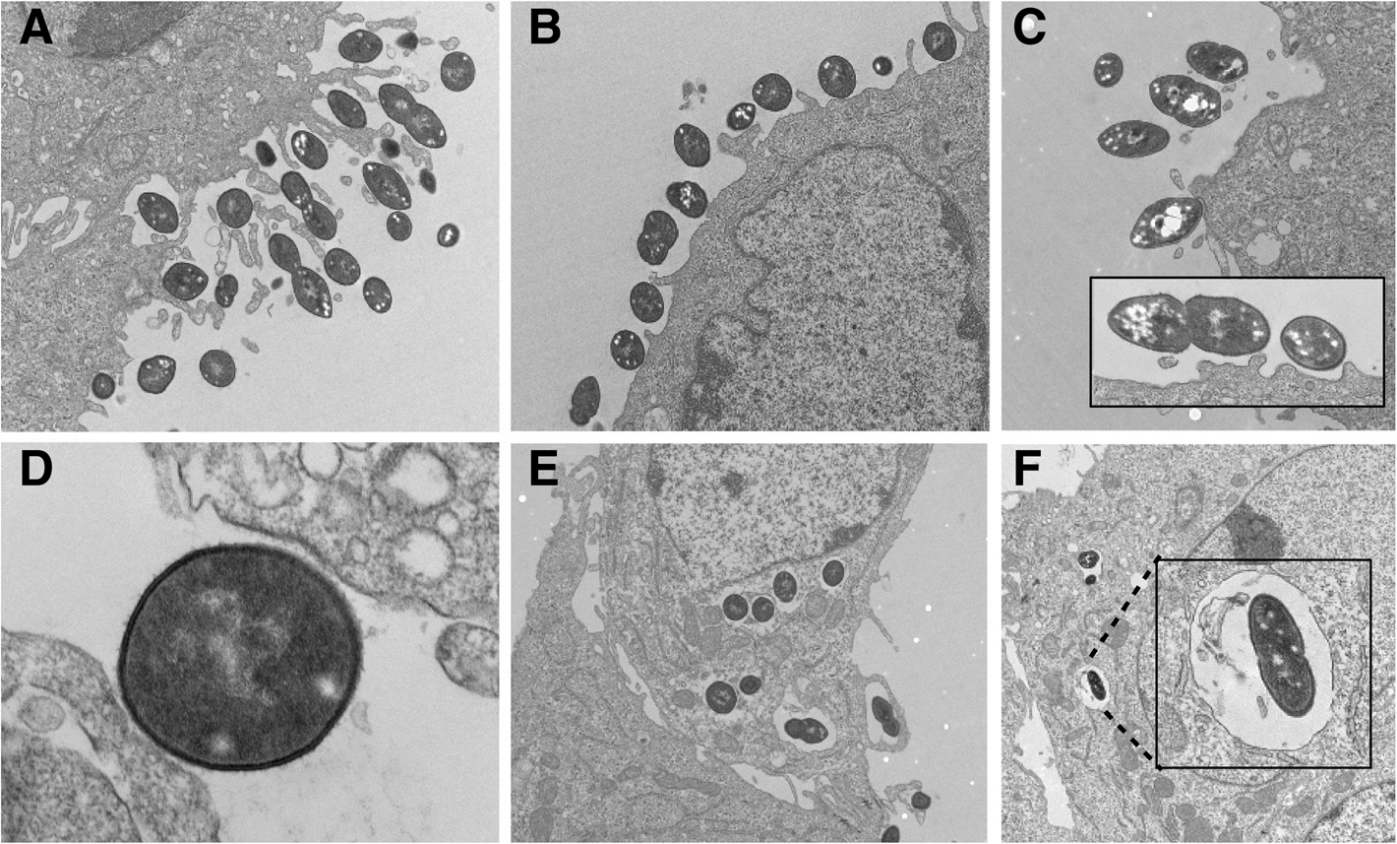
**Electron micrographs of D562 epithelial cells incubated with TIGR4 and G54 strains for 2 hours.** Pneumococci attach to the host cells either as micro-colonies (A - TIGR4) or single colonies (B, D - TIGR4, C (including inset) - G54) and are surrounded by host pseudopodia. Bacteria invade host cells and are internalized into cytoplasmic vacuoles (E - TIGR4 and F - G54). Original magnifications **A**, 4400X; **B**, 4400X; **C**, 4400X (inset, 15000X); **D**, 26000X; **E**, 3200X; **F**, 1650X (inset, 6500X).

These interactions were visualized using electron microscopy. Unlike G54, TIGR4 pneumococci were found in larger numbers adjacent to and within epithelial cells (Figure 1). Pneumococci attached to the monolayer of epithelial cells either as micro-colonies (Figure 1 A) or as single cells (Figure 1 B, C, D), and this was accompanied by the formation of elongations of the epithelial cell out towards and around the bacterial cells. The images indicated that pneumococci were internalized into host cell vacuoles (Figure 1 E, F).

**Figure 2:**
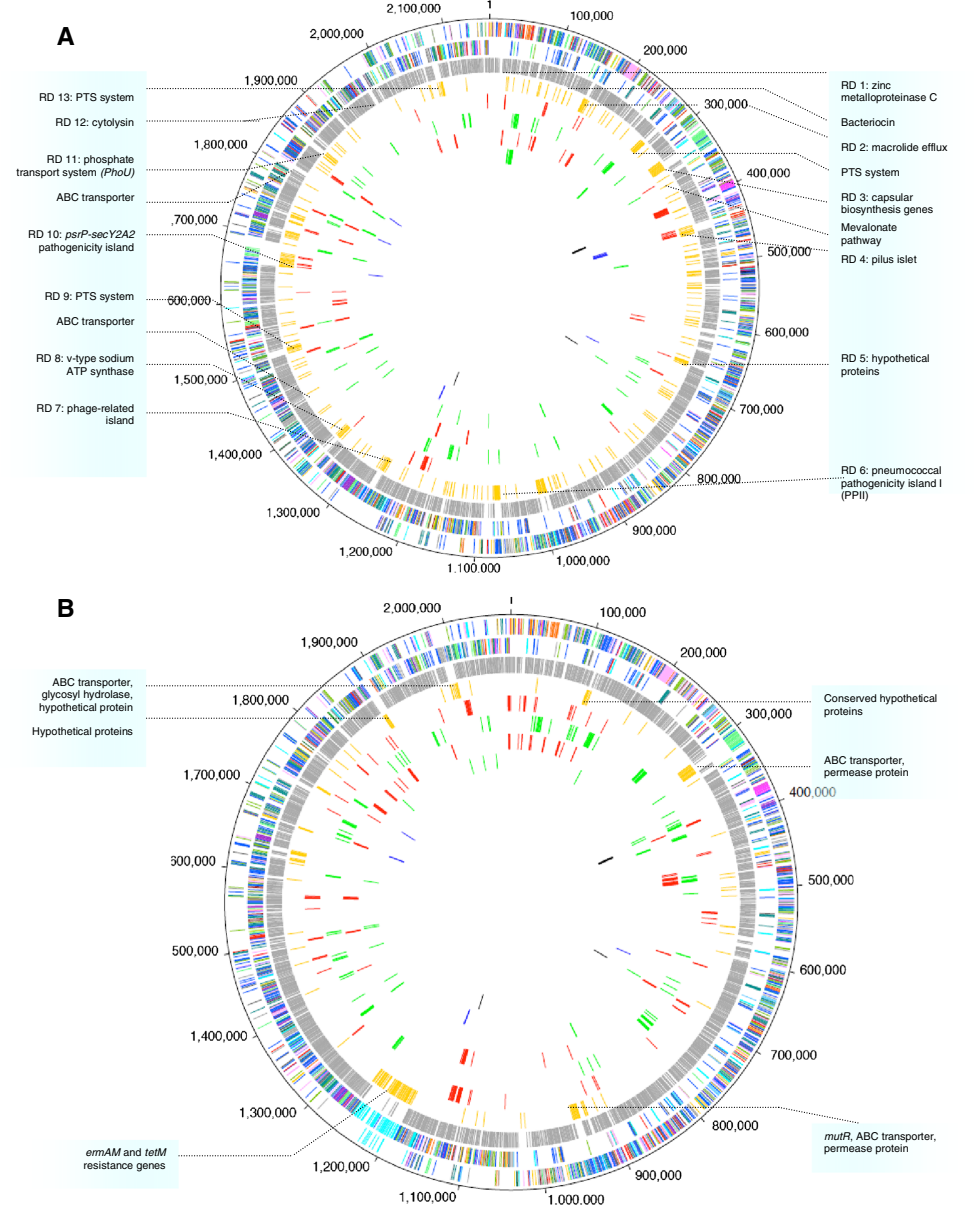
**Circular representation of the *S. pneumoniae *TIGR4 (A) and G54 (B) genomes and transcriptomes.** The two outermost circles show the predicted coding regions on the plus (outermost) and minus (2nd circle) strands color-coded by functional categories as in Tettelin *et al.*[60]. The third circle (light grey) shows genes that are shared by both TIGR4 and G54 genomes and the fourth circle (gold) shows strain-specific genes. Colored boxes depict genes in regions of diversity [61] as well as other strain-specific clusters (listed in Additional file 6). The red and green circles correspond to transcriptome results; fifth – genes up-regulated in adherent bacteria; sixth – genes down-regulated in adherent bacteria; seventh – genes up-regulated in non-adherent bacteria; eighth – genes down-regulated in non-adherent bacteria. The two innermost circles correspond to the genes selected for mutagenesis (asterisked loci in Table 1); blue – successfully knocked out (SP_0462-SP_0468, SP_0737, SP_1294-SP_1295, SP_1758, SP_1855 and SP_1922) and black – unsuccessful/not viable (SP_0423-SP_0427, SP_0783 and SP_1270).

Since TIGR4 and G54 differed significantly in their ability to adhere to and invade D562 cells, we sought to determine genetic differences between the strains. Direct BLASTP analysis (e-value cut-off of 10e^-15^) of TIGR4 and G54 proteins predicted from the whole genomes revealed that over 10% of each strain’s proteins are unique with respect to the other strain (15.7% TIGR4 and 12.4% G54). Representation of shared and unique proteins along the genomes revealed a number of strain-specific genomic islands encoding proteins potentially involved in the adherence and invasion processes (Figure 2, Additional file 6).

### Gene expression analyses

We analyzed the transcriptome of both pneumococcal strains upon exposure and adherence to human pharyngeal cells. DNA microarrays were used to analyze the gene expression profiles of *S. pneumoniae* in the following two ways: (i) transcription profiles of bacteria adhering to host cells were compared with those of bacteria grown in tissue culture medium in the absence of host cells (i.e., adherent bacteria vs. culture-medium-treated bacteria), and (ii) transcriptional profiles of adherent bacteria were compared to those of non-adherent bacteria exposed to the host cells (i.e., adherent bacteria vs. non-adherent bacteria). A schematic representation of the experimental setup is shown in Additional file 1.

**Figure 3:**
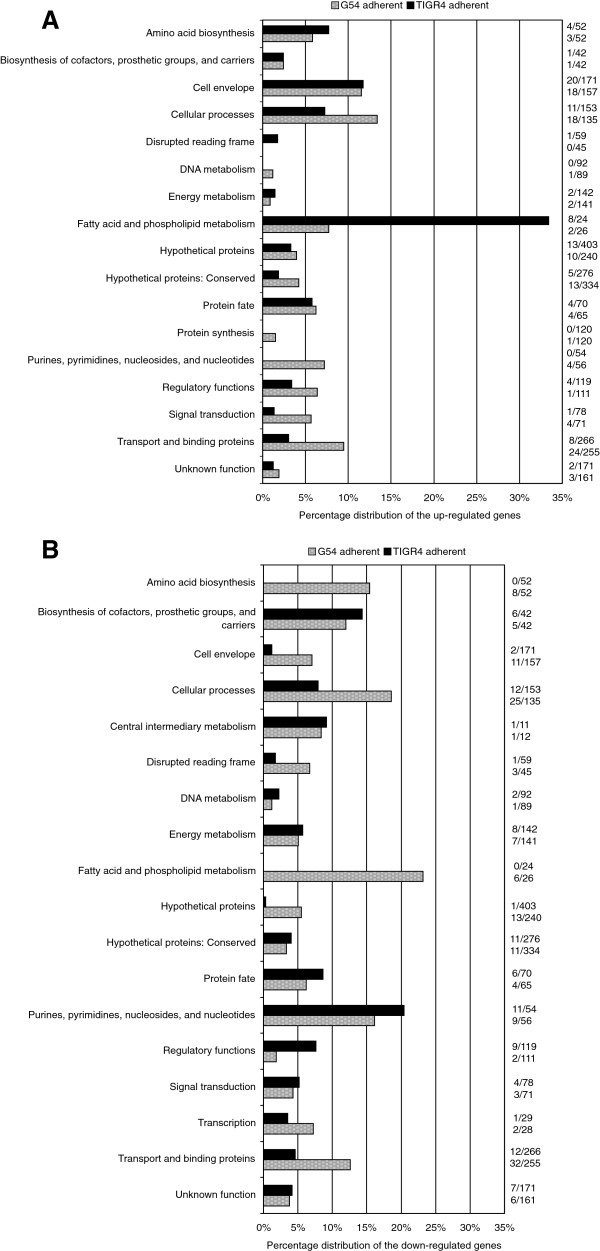
**Functional categories of differentially expressed genes in the adherent fraction of TIGR4 and G54. A**. Percentage distribution of the up-regulated genes, **B**. Percentage distribution of the down-regulated genes. TIGR4 genes are depicted as black bars and G54 genes as grey bars. The percentage of genes up- or down-regulated is based on the total number of genes in the genome assigned to a particular category. The numbers on the right show the fractions from where these percentages were derived.

*Analysis of gene expression in adherent bacteria compared to culture medium-treated bacteria:* After analysis of statistical significance, application of a 2-fold cutoff on expression ratios and addition of genes likely to be co-transcribed (see Methods), TIGR4 differentially regulated 154 genes while G54 regulated 242 genes (Additional file 7, Figure 2 – circles 5–8). The regulation ratios varied from 0.12-14.4. Eighty-four genes were commonly regulated by the adherent population of the two strains. Differentially expressed genes were classified into functional categories (Additional file 7). Categories that were significantly over-represented (Figure 3A and 3B) included ‘cellular processes’, ‘cell envelope’, ‘transport and binding’ and ‘fatty acid and phospholipid metabolism’ (*p* < 0.001; Fisher’s exact test, Bonferroni step down correction applied).

*Comparison of adherent and non-adherent pneumococci incubated in the presence of host cells:* We hypothesized that not only do bacteria remodel their transcriptome on sensing the presence of host cells, but that the degree of adjustment varies within the individual bacterial cells in the population eventually determining the outcome - successful attachment to host cells. Since we did not directly hybridize adherent bacteria against non-adherent bacteria on the same array, we compared the ratio of the adherent vs. control against the ratio of non-adherent vs. control (ratio index). This is mathematically equivalent to the ratio of adherent vs. non-adherent. To increase the sensitivity to meaningful functional differences between adherent and non-adherent bacteria as opposed to between adherent and culture medium-treated bacteria, we used an arbitrary cutoff of 1.5 fold change on the ratio index. Using this ratio cutoff and a statistical test of significance, 282 and 287 genes were differentially regulated in TIGR4 and G54, respectively. Of these, 79 were coordinately expressed (directionally) in both strains (Additional file 8). We infer that these commonly regulated genes are the basic set of genes associated with successful functional adherence.

Successful adhesion was associated with increases in the transcription of 412 genes in a strain-specific manner (Additional file 8). A few of the genes that were up-regulated in TIGR4 alone correspond to genes identified in the previous section and include: adhesins (*rlr* islet and glycosylated proteins), transporters (sodium SP_0737, amino acid SP_1502), fatty acid metabolism genes and several hypothetical proteins. Genes that code for hypothetical proteins were the most highly represented. These results provide a novel approach to the analysis of genes whose expression is important for intimate adherence and/or invasion of pneumococci to epithelial cells.

### qRT-PCR confirmation of gene regulation

**Figure 4:**
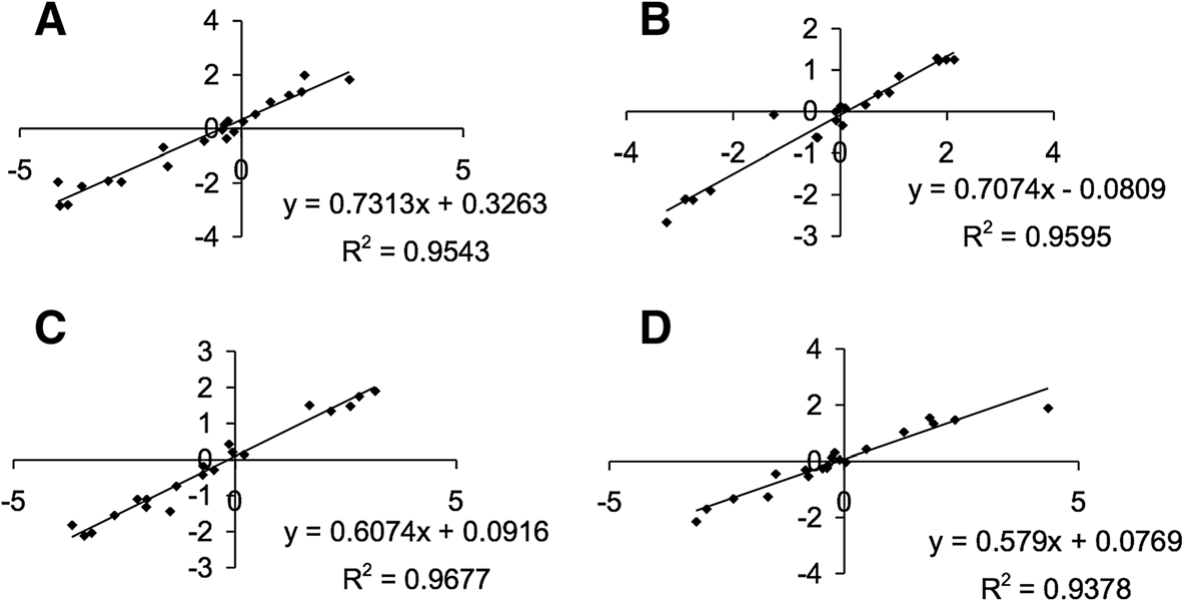
**Validation of microarray data by qRT-PCR.** mRNA levels of 21 genes obtained by microarray (y-axis) and qRT-PCR (x-axis) are plotted. **A**, TIGR4 adherent bacteria; **B**, TIGR4 non-adherent bacteria; **C**, G54 adherent bacteria; **D**, G54 non-adherent bacteria. Graphs show a positive correlation between microarray log_2_ ratios and qRT-PCR ΔCt.

A subset of 21 *S. pneumoniae* genes were selected for qRT-PCR validation of expression levels based on: i) a range of gene expression values i.e., up-, down- and non-regulated and ii) biological interest. The data were analyzed in separate groups for each strain and for adherent and non-adherent phenotypes. Each sample of each biological replicate was analyzed three times. In all four groups there was a strong positive correlation (correlation coefficient r > 0.968) between the microarray results and the qRT-PCR (Figure 4), supporting the validity of the microarray method. The qRT-PCR results from the TIGR4 adherent sample were further analyzed using the RNA-Seq method. Both techniques provide an absolute measure of the transcript abundance, with low abundance transcripts displaying a low average coverage in RNA-Seq and a high cycle threshold (Ct) in qRT-PCR. Additional file 9 shows the expected logarithmic correlation between the Ct observed in qRT-PCR and the average coverage observed in RNA-Seq for each gene. The complete list of RNA-Seq results for genes in the TIGR4 adherent sample are listed in Additional file 10.

### Adherence characteristics of wild type TIGR4 and isogenic knockout mutants

**Figure 5:**
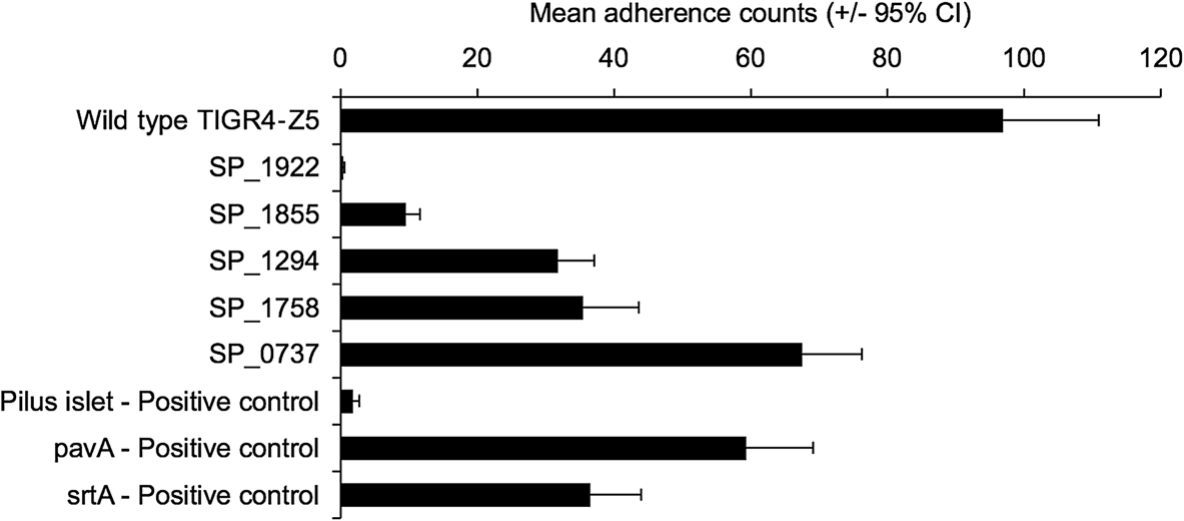
**Adherence of knockout mutants compared to the wild type strain.** The wild type and mutant strains were incubated for 2 hours with D562 cells and the number of adherent bacteria was determined. The adherence of mutants is given as mean adherence counts from 12 replicate wells (x-axis). Error bars are 95% confidence intervals observed among the replicates. All means differ significantly from the wild type at *p* < 0.001.

On the basis of the microarray results a list of the most highly attractive targets for further investigation was generated (Table 1). Of these, nine regions - 6 individual genes and 3 operons - were selected for knockout experiments (asterisked loci in Table 1, Figure 2, see Methods for prioritization criteria). Mutants were created using insertion-deletion mutagenesis whereby target genes were replaced with a kanamycin resistance-streptomycin sensitive cassette and then incorporated into the genome by genetic transformation and homologous recombination. Three mutants: SP_0783 (vitamin transporter), SP_1270 (alcohol dehydrogenase), and SP_0423 – SP_0427 (genes encoding the type II fatty acid synthesis pathway), were never obtained despite several attempts at transformation, suggesting that the genes might be essential for survival. The remaining six mutant strains exhibited a reduction in adherence to D562 cells when compared to the wild type strain (range: 30% to 99.7%) (Figure 5). Three of the mutants, the pilus pathogenicity islet (positive control), SP_1855, and SP_1922, were markedly attenuated in adherence. The adherence capacity of two other positive controls - Δ*srtA* and Δ*pavA* - was reduced by 60% and 40%, respectively. The SP_1294, SP_1758 and SP_0737 knockouts showed residual adherence. Growth of the mutants and wild type strain in EMEM were compared (Additional file 5) and the adherence phenotypes were independent of the growth rates of the mutants.

**Table 1:** List of genes prioritized for functional evaluation of their role in adherence

**Fold increase**						
		**TIGR4 adherent/**	**G54 adherent/**	**Conserved **^**§**^**(Avg. BLASTP Id.)**	**Predicted surface exposure**^**¶**^	
**Locus**	**Annotation**	**control**	**control**			**Reason for selection**
SP_0018	Conserved hypothetical protein	1.7	3.3	yes (98.4%)	no	Ratio index ≥ 1.5
SP_0024	Hypothetical protein	2.6	0.9	yes (98.1%)	no	Up-regulated in the more adherent strain
SP_0025	Hypothetical protein	2.7	1.1	yes (95.7%)	yes (TmHMM)	Up-regulated in the more adherent strain
SP_0026	Hypothetical protein	2.2	X	yes (78.8%)	yes (SP, TmHMM, AR)	Up-regulated in the more adherent strain
SP_0099	Hypothetical protein	2.2	1.2	yes (98.7%)	yes (TmHMM, AR)	Up-regulated in the more adherent strain
SP_0100	Conserved hypothetical protein	2.2	1.4	yes (99.3%)	no	Up-regulated in the more adherent strain
SP_0101	Putative transporter	1.4	1.9	yes (99.1%)	yes (SP, TmHMM, AR)	Co-opted as part of an operon
SP_0415	Enoyl-CoA hydratase	1.5	0.4	yes (99.3%)	no	Co-opted as part of an operon
SP_0416	Transcriptional regulator, marR family	1.7	X	yes (99.4%)	no	Co-opted as part of an operon
SP_0417	3-oxoacyl-(acyl-carrier-protein) synthase III	1.6	1.0	yes (99.6%)	no	Co-opted as part of an operon
SP_0418	Acyl carrier protein	1.8	0.9	yes (99.8%)	no	Ratio index ≥ 1.5
SP_0419	Enoyl-(acyl-carrier-protein) reductase	1.4	0.4	yes (99.4%)	no	Co-opted as part of an operon
SP_0420	Malonyl CoA-acyl carrier protein transacylase	1.9	0.6	yes (99.5%)	no	Co-opted as part of an operon
SP_0421	3-oxoacyl-[acyl-carrier protein] reductase	2.7	0.7	yes (99.4%)	yes (SP)	Up-regulated in the more adherent strain & Ratio index ≥ 1.5
SP_0422	3-oxoacyl-(acyl-carrier-protein) synthase II	2.4	0.7	yes (99.8%)	yes (SP)	Up-regulated in the more adherent strain
SP_0423*	Acetyl-CoA carboxylase, bitoin carboxyl carrier protein	3.6	0.9	yes (99.2%)	no	Up-regulated in the more adherent strain
SP_0424*	Similar to hydroxymyristoyl-(acyl carrier protein) dehydratase	3.6	1.0	yes (99.3%)	no	Up-regulated in the more adherent strain & Ratio index ≥ 1.5
SP_0425*	Acetyl-CoA carboxylase, biotin carboxylase	4.2	1.1	yes (99.9%)	no	Up-regulated in the more adherent strain & Ratio index ≥ 1.5
SP_0426*	Acetyl-CoA carboxylase, carboxyl transferase subunit beta	5.5	1.4	yes (99.9%)	no	Up-regulated in the more adherent strain & Ratio index ≥ 1.5
SP_0427*	Acetyl-CoA carboxylase, carboxyl transferase subunit alpha	2.5	1.6	yes (99.9%)	no	Up-regulated in the more adherent strain & Ratio index ≥ 1.5
SP_0462*	Transcriptional regulator, putative	2.1	NA	no (90.5%)	yes (SP, TmHMM, BCE)	Up-regulated in the more adherent strain & Ratio index ≥ 1.5
SP_0463*	Cell wall surface anchor family protein	2.6	NA	no (71.3%)	yes (SP, TmHMM, AR, LPxTG)	Up-regulated in the more adherent strain & Ratio index ≥ 1.5
SP_0464*	Cell wall surface anchor family protein	1.9	NA	no (98.3%)	yes (SP, TmHMM, AR, LPxTG)	Ratio index ≥ 1.5
SP_0465*	Cell wall surface anchor family protein	2.1	NA	no	no	Up-regulated in the more adherent strain & Ratio index ≥ 1.5
SP_0466*	Sortase, putative	2.0	NA	no (98.6%)	yes (TmHMM)	Up-regulated in the more adherent strain & Ratio index ≥ 1.5
SP_0467*	Sortase, putative	2.3	NA	no (96.0%)	yes (TmHMM)	Up-regulated in the more adherent strain & Ratio index ≥ 1.5
SP_0468*	Sortase, putative	2.2	NA	no (95.8%)	yes (SP, TmHMM, AR, LAS)	Up-regulated in the more adherent strain & Ratio index ≥ 1.5
SP_0617	Conserved domain protein	1.5	3.6	yes (97.8%)	yes (SP, TmHMM)	Ratio index ≥ 1.5
SP_0737*	Sodium-dependent transporter	2.4	X	yes (96.3%)	yes (SP, TmHMM)	Up-regulated in the more adherent strain & Ratio index ≥ 1.5
SP_0738*	Conserved domain protein	2.0	X	yes (99.2%)	no	Up-regulated in the more adherent strain & Ratio index ≥ 1.5
SP_0783*	Conserved hypothetical protein	1.5	1.6	yes (96.3%)	yes (SP, TmHMM, AR)	Ratio index ≥ 1.5
SP_0879	Hypothetical protein	2	3.9	yes (99.2%)	yes (SP, BCE, AR)	Up-regulated in both strains & Ratio index ≥ 1.5
SP_1003	Conserved domain protein	0.8	3.9	yes (73.3%)	yes (SP, BCE, AR)	Up-regulated in the less adherent strain and downregulated in more adherent strain
SP_1127	Hypothetical protein	1.4	1.6	yes (98.6%)	no	Ratio index ≥ 1.5
SP_1256	Conserved hypothetical protein	X	1.9	yes (96.7%)	yes (TmHMM)	Ratio index ≥ 1.5
SP_1270*	Alcohol dehydrogenase, zinc containing	3.1	3.6	yes (99.1%)	no	Up-regulated in both strains
SP_1294*	Crcb protein downstream of a putative chorismate mutase	2.0	1.1	yes (98.6%)	yes (SP, TmHMM)	Up-regulated in the more adherent strain
SP_1295*	Crcb protein downstream of a putative chorismate mutase	1.8	1.1	yes (98.1%)	yes (TmHMM)	Co-opted as part of an operon
SP_1600	Putative membrane protein	2.0	2.0	yes (99.2%)	yes (SP, TmHMM)	Up-regulated in both strains
SP_1601	Conserved hypothetical protein	2.0	2.0	yes (99.0%)	yes (SP, TmHMM)	Up-regulated in both strains & Ratio index ≥ 1.5
SP_1602	Required for expression of the phosphonate utilization phenotype in *E. coli*	2.5	2.1	yes (99.0%)	no	Up-regulated in both strains & Ratio index ≥ 1.5
SP_1758*	Glycosyl transferases in the psrP-secY2A2 pathogenicity island	2.2	X	no (86.3%)	no	Up-regulated in the more adherent strain
SP_1855*	Dehydrogenase	6.0	1.0	yes (99.7%)	no	Up-regulated in the more adherent strain
SP_1856	Transcriptional regulator, merR family	7.6	1.1	yes (99.7%)	no	Up-regulated in the more adherent strain
SP_1857	Cation efflux system protein	14.4	X	yes (98.1%)	yes (SP, TmHMM)	Up-regulated in the more adherent strain
SP_1922*	Conserved hypothetical protein	2.3	1.0	yes (99.8%)	yes (BCE)	Up-regulated in the more adherent strain & Ratio index ≥ 1.5
SP_1923	Pneumolysin	3.0	0.5	yes (99.8%)	no	Up-regulated in the more adherent strain
SP_1924	Hypothetical protein	2.6	0.4	yes (99.0%)	no	Up-regulated in the more adherent strain
SP_1925	Hypothetical protein	2.3	0.4	Yes (98.2)	no	Up-regulated in the more adherent strain

*Loci selected for knockout experiments, either individually or as an entire operon.

^**§**^Conserved = present in all pneumococcal loaded in the Strepneumo Sybil system [24]; Avg. BLASTP Id. = % of the characters in the alignment that match the conserved sequence, including gaps.

^**¶**^SP = signal peptide; TmHMM = trans-membrane spans; BCE = B-cell epitopes from BepiPred; AR = antigenic regions from EMBOSS antigenic; LPxTG = proteins covalently linked to peptidoglycan; LAS = lipoprotein attachment site.

### Characterization of SP_1922

**Figure 6:**
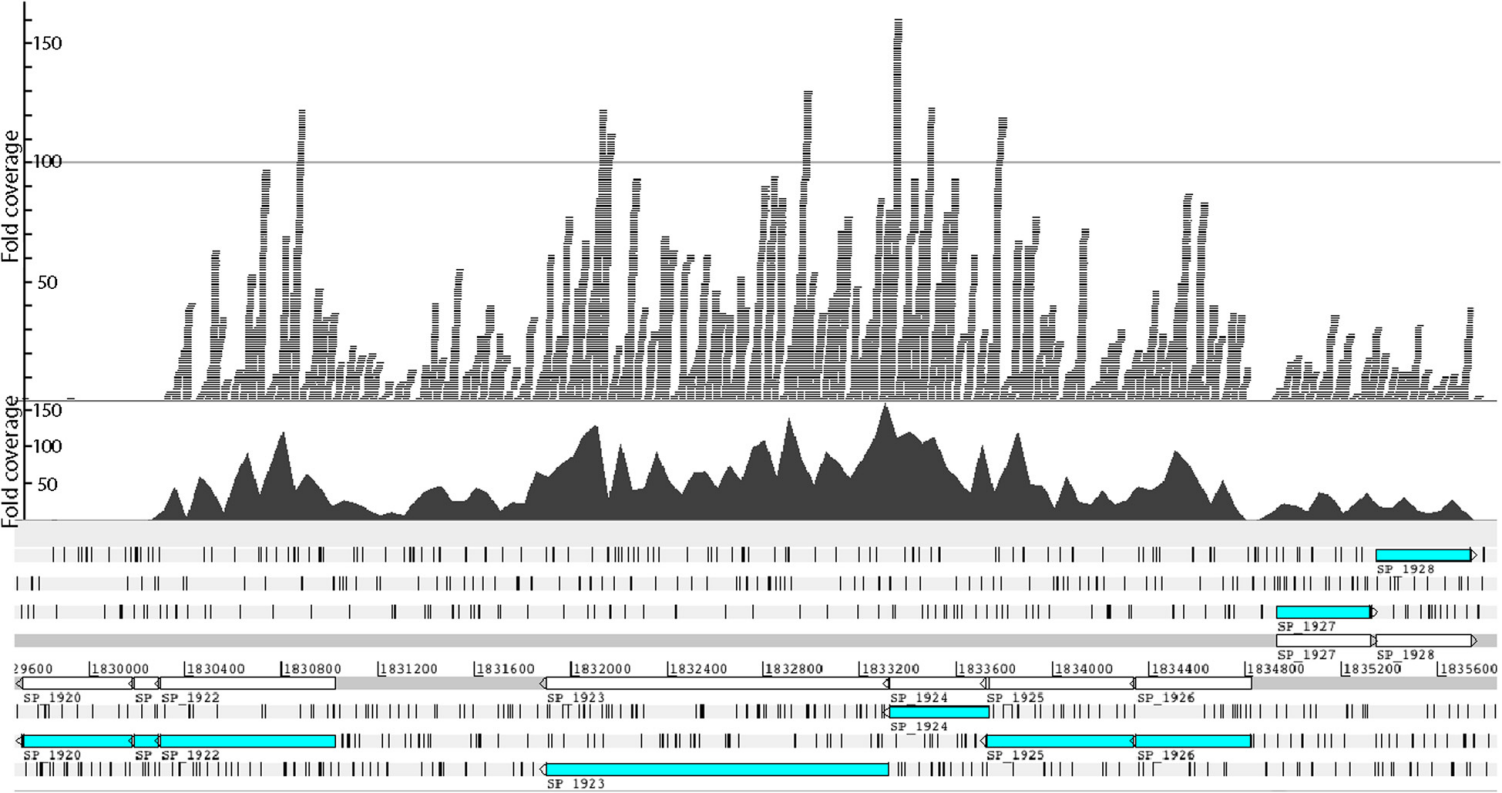
**Characterization of SP_1922.** RNA-Seq expression coverage of SP_1922 and neighboring regions drawn using Artemis [62]. RNA-Seq reads from TIGR4 adherent samples were mapped onto the TIGR4 genome. Predicted genes are shown in cyan on the bottom panel that displays the six frames of translation. Grey bars on the top panel represent RNA-Seq reads and the black plot below them represents the amount of read coverage, which is proportional to expression levels. The coverage plot across genes SP_1922 - SP_1926 is uninterrupted, indicating that these genes are likely to be expressed as an operon.

The ΔSP_1922 mutant was the most thoroughly attenuated in adherence (99.7%). SP_1922 encodes a hypothetical protein that is present in all pneumococcal genomes published to date. The protein length (238 aa), location, and sequence is identical (99.9 - 100%) in all of the sequenced pneumococcal genomes presented in the Strepneumo Sybil system ([24], see Table 1 for a comparison of the average identity of other genes). By BLASTP analysis (e-value cut-off of 10e^-15^), this gene is conserved in all streptococcal species and is a core gene in several other bacterial species e.g., *Moraxella catarrhalis*, *Escherichia coli, Salmonella enterica,* and *Shigella sp*. Conservation of the gene suggests that it is an important bacterial protein, yet its function is unknown. SP_1922 in *S. pneumoniae* is located immediately downstream of the pneumolysin gene (*ply*), which encodes a major pneumococcal toxin that has been implicated in adherence and invasion in several studies [3,25-28]. There are a number of repeat elements (BOX elements and tandem repeats) in the intergenic region between *ply* and SP_1922. This region appears to be unique to pneumococci. SP_1922 is predicted by the BepiPred software ([29], threshold 2.2) to carry a B-cell epitope. Our microarray results showed that SP_1922 – SP_1926 were all up-regulated in TIGR4 upon contact with host cells and down-regulated in the less adherent G54 strain. Illumina-based RNA sequencing (RNA-Seq, reviewed in [30]) data was generated from TIGR4 adherent samples (36 nt unpaired reads). RNA-Seq coverage revealed that SP_1922 is most likely transcriptionally linked to *ply* (SP_1923) in a manner consistent with genes organized in an operon (Figure 6, Additional file 10).

**Figure 7:**
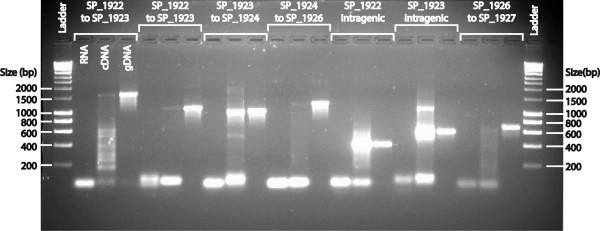
**DNA fragments derived from various amplicons of the region spanning SP_1922 – SP_1926.** RT-PCR on *S. pneumoniae* strain TIGR4 grown in rich medium (THYE) showed amplification products of the expected sizes. Lane 1 = Hyperladder I (Bioline). The remaining lanes represent pairs of primers tested on (i) unprocessed RNA (negative control, confirms absence of contaminating gDNA), cDNA (query), and gDNA (positive control), all from strain TIGR4. Primer pairs spanned: SP_1922 and SP_1923 (*ply)* (lanes 2–4 & 5–7), SP_1923 and SP_1924 (lanes 8–10), SP_1924 and SP_1926 (lanes 11–13), SP_1922 intragenic region (lanes 14–16), SP_1923 intragenic region (lanes 17–19), and SP_1926 and SP_1927 (negative control, lanes 20–22). It should be noted that the band of the highest molecular weight in the cDNA lane of the negative control (lane 21) is smaller than the band in the gDNA lane (22) and is part of a non-specific smear of amplification.

To confirm this prediction, the five-gene operon was analyzed with reverse transcriptase PCR (RT-PCR). Using primers spanning various regions within the operon (Additional file 4), RT-PCR showed that hypothetical proteins SP_1922, SP_1924, SP_1925 and SP_1926, were transcribed in an operon with the gene encoding pneumolysin (SP_1923) (Figure 7). Due to paucity of the original TIGR4 adherent RNA samples, the RT-PCR experiments were performed on RNA samples from TIGR4 grown in THYE medium.

## Discussion

Adherence of *S. pneumoniae* to the human nasopharynx is a critical process and first step that enables the pneumococcus to survive and infect the host. In some cases, the adherent pneumococci penetrate the mucosal epithelium of the nasopharynx and cause systemic disease. The factors that facilitate the transition from adherence to internalization are not well known. We examined the adherence and invasion of pneumococci by using a human pharyngeal epithelial cell line, D562, which is an *in vitro* model for adherence and for the purpose of this study, mimics the natural site of pneumococcal colonization. Our results show that *S. pneumoniae* TIGR4 strain was more capable of adhering to and being internalized within epithelial cells than G54. This difference was not due to differences in growth patterns, susceptibility to gentamicin, or the G54 strain being more toxic to D562 cells since the EMs showed healthy cells and intact monolayers. Internalization, however, appeared to occur via a similar route in the two strains. Large vacuoles containing pneumococci were observed after infection with either strain. This observation opens up the possibility that pneumococci (or some pneumococcal strains) are not strictly extracellular pathogens as has been traditionally assumed. In support of this finding, Peppoloni *et al.* showed that the encapsulated strain TIGR4 was protected from intracellular killing, albeit in a different cell line, murine microgial cell line BV2 [31]. The same observation was made by Briles *et al.* who found TIGR4 embedded within granulocyte-filled nasal crypts in the sub-mucosa [32]. Rajam *et al.*[17] demonstrated that the internalization of three pneumococcal strains was in the range of 20-29%, a figure that is comparable to what we found in our study. Our results differ from that of the seminal work by Cundell *et al.*[33], who showed low invasion capacity of pneumococci. It is worth noting that the invasion assays were performed on a different cell line (primary human umbilical vein endothelial cells vs. D562 cells used in this study), a lower gentamicin concentration (50 μg/ml vs. 200 μg/ml), and the time before gentamicin addition was also significantly shorter (only 30 min) perhaps minimizing internalization. The observation that *S. pneumoniae* invasion is cell-type specific has been previously documented [34], with D562 cells exhibiting extensive invasion compared to other cell types.

The phenotypic differences in TIGR4 and G54 can be explained, at least in part, by differences in their genomes and transcriptomes. Genomic differences between the two strains were mainly located in the regions of genomic diversity (RD) (Figure 2). For example, TIGR4, which was superior in adherence and invasion, contained pathogenicity islands *rlr* and *psrP-secY2A2.* Previous studies have shown that strains lacking the *rlr* islet were attenuated in their ability to adhere to eukaryotic cells and to cause invasive disease [35,36] and presence of the *psrP-secY2A2* gene cluster has been shown to correlate with the propensity of bacteria to cause invasive pneumococcal disease [37,38]. These loci and the others (Additional file 6) may therefore be relevant to TIGR4’s interaction with the host but are dispensable in the G54 strain. The strain-specific contribution of certain genes to virulence has been demonstrated in other studies [39-43]. Deletion of *cbpA* gene from the serotype 4 TIGR4 strain reduced its virulence in a mouse pneumonia model; but had no effect on strains of serotypes 2, 3 and 19F [40]. Similarly, deletion of *ppmA* (putative proteinase maturation protein A) in a mouse colonization model reduced adherence in TIGR4 and a serotype 35 strain NTC10319 but not in a serotype 2 strain, D39 [39].

Analysis of the transcriptome of TIGR4 and G54 revealed the following biological themes as being the most pronounced:

G*enes encoding cellular processes.* Genes *comA-E*, *adcA-C,* and *adcR,* involved in inducing competence [44], were all significantly up-regulated in G54 on contact with host cells but only slightly up-regulated in TIGR4. Eleven bacteriocin-associated genes were also regulated in this strain, contributing to the high number of down-regulated genes encoding cellular processes. Bacteriocin-encoding genes have previously been shown to be co-regulated with competence genes in some studies [45,46] but not in others [47]. Pneumolysin *(ply)* was up-regulated in TIGR4 but down-regulated in G54. The toxin is important for disrupting the tight junctions between epithelial cells and allowing a portal of entry for pneumococcal invasion. Data from Thornton *et al.*[48] showed that *ply* induces transcription of host ICAM-1, an epithelial cell adhesion molecule that plays a role in pneumococcal adherence. *Ply* mutants have been shown to be attenuated in their ability to colonize the nasopharynx in mice [25]. In addition*, ply* mRNA was shown to be more abundant in the nasopharynx than in blood using two pneumococcal strains, D39 (serotype 2) and WCH16 (serotype 6A) [3,27]. Recent data from Price *et al.* revealed that Ply is localized to the cell wall in a LytA-independent manner [49]. Given the highly efficient attachment and invasion of epithelial cells by TIGR4 compared to G54 in our experiments, we speculate that the strain-specific expression of *ply* might be partly responsible for the observed phenotypic differences, hence supporting the role of pneumolysin in adherence and invasion. The unique up-regulation of *ply* in TIGR4 and the preferential up-regulation of quorum sensing systems in G54 suggest two different strategies for colonization in the nasopharynx that are consistent with the surface changes depicted by the electron micrographs (Figure 1). An alternative interpretation, which cannot be differentiated in these experiments, is that there are no strain-specific differences but that the observations made represent different stages of cellular infection. If this is the case, the progression through stages appears more rapid for TIGR4.

Interestingly, four hypothetical genes located immediately upstream (SP_1924, SP_1925, SP_1926) and downstream (SP_1922) of the *ply* gene were expressed in exactly the same manner as *ply* in both strains suggesting that they might be transcriptionally linked. We performed RT-PCR experiments to confirm expression of SP_1922-SP_1926 as a single operon (Figure 7). Further, Yadav *et al*. [50] have shown that SP_1924 and SP_1925 are implicated in the transport of Ply from the cytosol to the cell wall compartment, supporting the likelihood of their co-expression. The four hypothetical genes in the *ply* operon represent interesting candidates for further study to determine their exact role in adherence. Whether or not the BOX elements around this region control the expression of this operon is also a matter worth exploring in the future.

*Genes belonging to the cell envelope category.* There was enhanced expression of the *lic* operon in both strains as has been shown by Orihuela *et al.*[4], suggesting that bacteria were increasing phosphorylcholine levels on their cell wall, thereby enhancing adhesive interaction with host cells. TIGR4 up-regulated the *rlr* pathogenicity islet genes encoding pneumococcal pili, as well as the glycosylated proteins within RD10 (Additional file 6). Genes in these regions have previously been shown to have adherence functions [11,35,36,51]. There were considerable differences in expression in sub-populations of bacteria within the same strain; pili, *lic* and *psrP-secY2A2* operons were more highly expressed in the adherent sub-population as compared to the non-adherent sub-population, when in the presence of host cells.

*Genes encoding transport and binding proteins.* Genes belonging to the *psa* operon, a manganese ABC transporter system, were significantly up-regulated in both strains. These genes have previously been implicated in pneumococcal pathogenesis, specifically adhesion [4,6,8,52,53]. *phnA*, encoding an alkylphosphonate utilization protein transporter, as well as a gene encoding a putative membrane transporter (SP_1600), and a hypothetical gene (SP_1601) downstream of it were up-regulated in both strains. In *E. coli*, the *phn* operon consists of 14 genes that are regulated by the phosphate (*pho*) system [54]*.* Induction of *phn* genes in our study suggests that overcoming phosphate limitation might be important for adherence and invasion. Interestingly, *adhC* (SP_1855), SP_1856, and *czcD* (SP_1857) encoding a zinc-containing alcohol dehydrogenase, a MerR family regulator, and a zinc efflux pump, respectively, were significantly up-regulated in TIGR4. AdhC and the MerR family regulator have previously been shown to be important for virulence [55]. In a separate study [56], these genes appeared to be required for resistance to nitric oxide stress, but did not play a key role in nasopharyngeal colonization *in vivo*. Nitric oxide is recognized as a key component of the innate immune response. Nitric oxide can inhibit bacterial DNA replication via the release of zinc from metalloproteins and can also hinder bacterial respiration. Hence bacteria e.g. pneumococci, *Haemophilus influenzae* and *E. coli* have developed nitric oxide resistance mechanisms such as the *adhC*-*czcD* system [56-59]. Expression of these genes in our study is consistent with our earlier observation that a sub-population of bacteria penetrated the epithelial cells; the observed regulation might be an adaptation to the intracellular environment. It is conceivable that induction of *ply* by TIGR4 resulted in epithelial damage, which in turn led to triggering of nitric oxide production by the host cells, placing bacteria under nitrosative stress. *AdhC* (SP_1855) is up-regulated to reduce the nitric oxide, therefore allowing internalized bacteria to resist killing.

*Other regulated genes.* Genes encoding metabolism-associated proteins were differentially expressed upon adherence of pneumococci to D562 cells. These include genes involved in amino acid biosynthesis, nucleotide, sugar, fatty acid and phospholipids metabolism. A number of the differentially regulated genes encoded hypothetical proteins or proteins with unknown functions. Many of these proteins are predicted to be >100 aa (low probability to arise from randomly occurring, spurious open reading frames) and have signal peptides or other motifs indicating that they are likely to be functional surface localized proteins (Table 1). These properties make them good candidates for further characterization of their utility as vaccine antigens.

Our mutagenesis data showed that deletion of both SP_1922 (encoding a conserved hypothetical protein) and the *rlr* islet, independently resulted in almost complete attenuation of adherence. Though we expected the *rlr* mutant to be attenuated in adherence, the extent to which adherence was reduced (98%) was surprising since it is known that strains without pili bind to epithelial cells, albeit less efficiently [35]. It is conceivable that SP_1922 and *rlr* might act in synergy or might be co-regulated and therefore deletion of one gene affects the function of the other. It has previously been shown that adherence via pili is coordinated with adherence by other adhesins [36]. Deletion of the whole pilus islet in our study might have affected the regulation of some genes (coding for adhesins) outside the islet, SP_1922 included. On the other hand, deletion of SP_1922 totally abolishes all the adhesion seen in this *in vitro* model. Of note, the knockout was carefully constructed to prevent interfering with *ply*, which is upstream of this gene. In addition, the activity of pneumolysin in the SP_1922 mutant was confirmed to be similar to that of the wild type using a hemolytic activity assay (Additional file 11). We are tempted to speculate that this gene encodes the most important adhesin in this *in vitro* assay system. Deletion of this gene probably changes an important adhesive property without which other adhesins cannot function. Further investigations are needed to elucidate our findings.

## Conclusion

We have identified a novel role for several pneumococcal genes in adherence to pharyngeal epithelial cells. This work therefore identifies a short list of genes that encode proteins that can be further evaluated as potential antimicrobial targets or non-capsular vaccine candidates if immunogenicity can be demonstrated in future studies.

## Abbreviations

D562 cells: Detroit 562 cells; RNA-Seq: RNA sequencing; Reverse transcriptase PCR: RT-PCR; SAM: Significance analysis of microarrays; Ply: Pneumolysin; RD: Regions of genomic diversity; PsaA: Pneumococcal surface adhesion A; PsrP: Pneumococcal serine repeat protein; CbpA: Choline binding protein A.

## Competing interests

JAGS reports receiving a grant from GlaxoSmithKline Biologicals (Anthony Scott, Kayla Laserson; $2,575,975; Oct 2010-Sep 2013) for a study entitled: ‘A phase IV multi-site observational epidemiology study to assess potential risk for adverse events following immunization that may be associated with misuse of a two-dose vial of 10-valent Pneumococcal Conjugate Vaccine (Synflorix) in Kenya’.

## Authors’ contributions

All authors read and approved the final manuscript. SZKM carried out the adherence and invasion assays, prepared cells for electron microscopy, performed the pneumococcal microarray experiments and knockout experiments, analyzed the data, and drafted the manuscript. SRS participated in the design and performance of bacterial adherence and invasion assays, electron microscopy, RNA extractions and drafting of the manuscript. JCDH participated in the design and analysis of all experiments involving molecular work in this manuscript. NK performed the PCR for confirmation of operon structure and qRT-PCR experiments. NI participated in the pneumococcal microarray experiments. DRR participated in the bioinformatics analysis of microarray work. UF, THC, LJT, XL participated in the RNA sequencing. CSG performed the electron microscopy. JS and GMC participated in the design of the study. SKH participated in the design and performance of knockout experiments. JAGS and HT conceived the study, participated in the study design and development, analysis, and drafting of the manuscript.

## Supplementary Material

Additional file 1 Is a figure showing an overview of the microarray experimental set up. Click here for file

Additional file 2 Is a table listing the primers used for qRT-PCR analysis of bacterial genes. Click here for file

Additional file 3 Is a table listing the primers used for knockout mutagenesis. Click here for file

Additional file 4 Is a table listing the primers used for RT-PCR confirmation of the pneumolysin operon. Click here for file

Additional file 5 Shows growth curves in EMEM of G54 and TIGR4 wild type strains, as well as the TIGR4 isogenic mutants. Click here for file

Additional file 6 Is a table listing the regions of diversity between TIGR4 and G54 strain. Click here for file

Additional file 7 Is a table listing genes differentially expressed in cell-adherent pneumococci vs. culture medium control pneumococci. Click here for file

Additional file 8 Is a table listing genes differentially expressed in cell-adherent pneumococci vs. non-adherent pneumococci exposed to D562 pharyngeal cells. Click here for file

Additional file 9 Is a figure showing the correlation between RNA-Seq average coverage and qRT-PCR thresholds. Click here for file

Additional file 10 Is a table showing the complete list of RNA-Seq results. Click here for file

Additional file 11 Is a table showing the hemolytic activity of pneumolysin in TIGR4 wild type and its SP_1922 isogenic mutant strain. Click here for file
