# Supplementary material for: Phenotypic, genomic, and transcriptional characterization of Streptococcus pneumoniae interacting with human pharyngeal cells
Source: BMC Genomics. 2013 Jun 9;14:383. doi: 10.1186/1471-2164-14-383 (PMC3708772; doi:10.1186/1471-2164-14-383)
Supplement: Additional file 1 — Is a figure showing an overview of the microarray experimental set up. [file 1471-2164-14-383-S1.pdf]

Additional data file 1. Overview of microarray experimental set up.

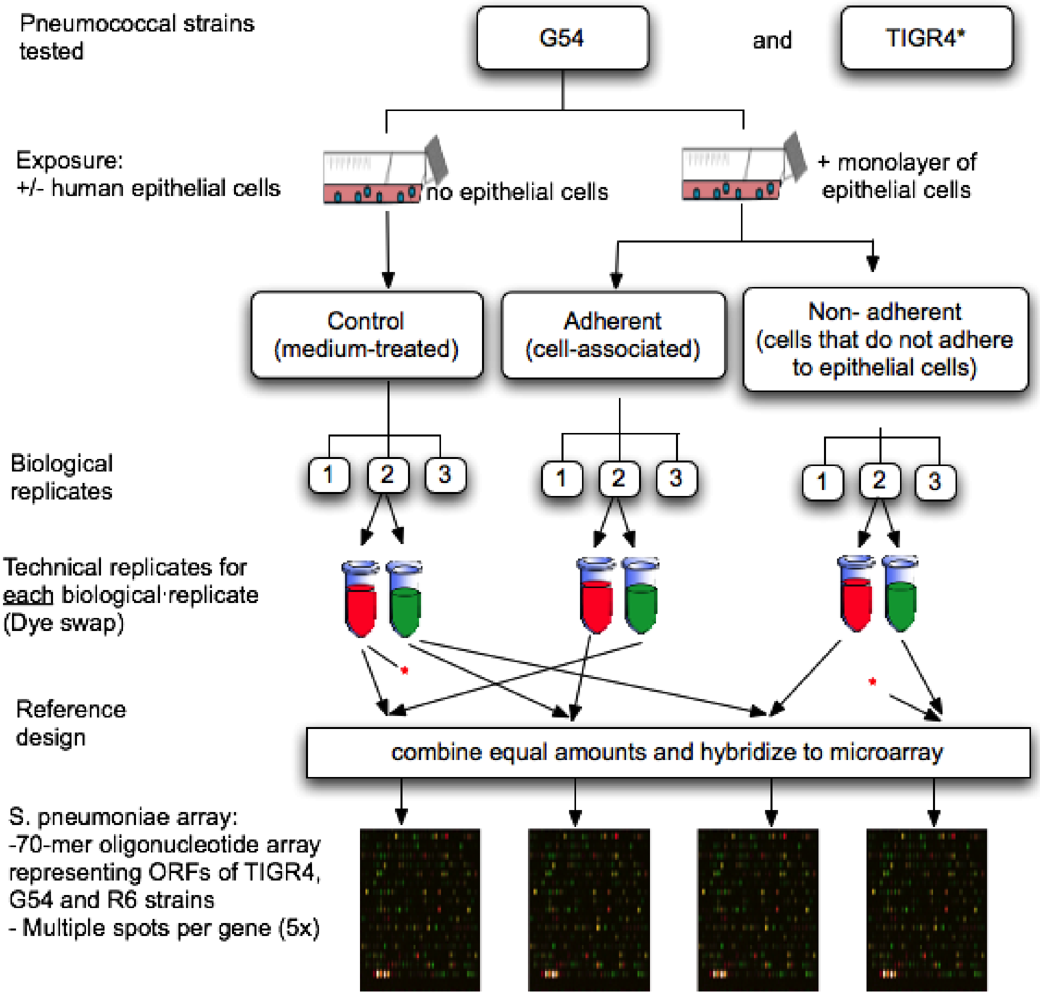

*The same experimental design was applied to the TIGR4 strain
