# Supplementary material for: Phenotypic, genomic, and transcriptional characterization of Streptococcus pneumoniae interacting with human pharyngeal cells
Source: BMC Genomics. 2013 Jun 9;14:383. doi: 10.1186/1471-2164-14-383 (PMC3708772; doi:10.1186/1471-2164-14-383)
Supplement: Additional file 2 — Is a table listing the primers used for qRT-PCR analysis of bacterial genes. [file 1471-2164-14-383-S2.pdf]

Additional data file 2. List of primers used for qRT-PCR analysis of bacterial genes.

Locus/gene symbol	F-Primer	R-Primer	Prod Size
SP_1810	TCCTTTCCAATTCAGATGGAG	CGTGCATCAGGCATAACAAC	158
SP_2157	GCGATTGCTGAGATTGAGAAG	AGGCATCAATCAAGGCATTC	124
SP_1923/*ply*	CAGTCGCCTCTATCCTGGAG	AGCCAACAAATCGTTTACCG	196
SP_0463	TCACGGGAATACTCCAAAGC	CAAATCGAACGTTGCTTCAG	121
SP_2197	AGAATACGCAAGCGACAAGG	ATTCGTTGGTGAAGCCTTTG	128
SP_0175	CTAAGGGAATCGCCCAAATC	CCTTGTGCACACTCAGAACC	136
SP_1987	AACGTTTGGAAAGGCAAGTG	GTCTTAGCAAGGGCAACTCG	118
SP_0238	AATCTGGAATTGTTGCAGGTG	CTAGATACAACAGCCATCATCG	115
SP_1511	CAGGTTCTGGCCTACAATCAC	TCAAAGGGAGCAAACGAGTC	154
SP_1499	TCGTAGCTGAAACCAAAGCTC	GACATTGAAGAGTCGCAACG	163
SP_1648/*psaA*	AGGTCAGGCATTTCTCGATG	AGACGCATTCCTTGACCTTG	116
SP_1649/*psaB*	TCCTGATTGTAGCCATGCTG	ACCTGCCGCAACATTAAAAC	152
SP_1650/*psaC*	AACAATTGAGCGCCAAAGAC	AATGCTCCTTCGCTGGTTAC	157
SP_1267/*licC*	GCAATGATTTGACACGTTCG	ACCACTAAGGATGCGACCTG	137
SP_2135	AACACAAAGAATCTGGTGAACG	AACACAACGTGTTTGCGAAG	112
SP_0002	TTCCTAGCCGTTCTCTACGC	TGTTGGAATCAAGCGATCTG	167
SP_0085	GGACCATCTTGGAAACAAGC	GCCAATTGCAAACCGTATTC	140
SP_0044	CGTGCTCCGCAACTATACTG	CAAGTAGGCAATCTGCTGGTC	181
SP_0784	TGTAACGGGCGAGAAAGAAC	AGTTCCGATAGCAGGGTGTG	145
SP_1587	TTTAACAGCTTGGGCAATGG	AGACAAAGCCAAGGCGATAC	130
SP_1588	TGTTGCTGGTACAGGCGTAG	AAGTCCAGGGATTGGCAAG	160
