# Supplementary material for: Phenotypic, genomic, and transcriptional characterization of Streptococcus pneumoniae interacting with human pharyngeal cells
Source: BMC Genomics. 2013 Jun 9;14:383. doi: 10.1186/1471-2164-14-383 (PMC3708772; doi:10.1186/1471-2164-14-383)
Supplement: Additional file 3 — Is a table listing the primers used for knockout mutagenesis. [file 1471-2164-14-383-S3.pdf]

Additional data file 3. List of primers used for knockout mutagenesis.

Primer name*	Sequence (5' to 3')**	Restriction site
SP1758_gtf_J1	GGG GCT TGA TTG TTA TTG GGA C	
SP1758_gtf_J2	CA<u>**G GTA CC**</u>A GTC ACG ATT TCA CCT TTT GGA GTG	KpnI
SP1758_gtf_J3	CA<u>**G AGC TC**</u>G CAG ATA GCG AGC CGA TTA TGA C	SacI
SP1758_gtf_J4	TCA ACC AAA GCA GCA ACT TCA CTA C	
SP0737_Na_J1	TAG CCT CGT CTT CGT GAC CT	
SP0737_Na_J2	CA<u>**G GTA CC**</u>C CCA TTG TGA TTT TTC TG	KpnI
SP0737_Na_J3	CA<u>**G AGC TC**</u>T TAA CAG TTA CAG GAC GGC TA	SacI
SP0737_Na_J4	TTT GCT ATA ATG AGG GGA GA	
SP1294_Na_J1	TCT GGT TTC ATA TGA CGA TG	
SP1294_Na_J2	CA<u>**G GTA CC**</u>A CAG TCT CCT CCT TTA TTT GA	KpnI
SP1294_Na_J3	CA<u>**G AGC TC**</u>A AAT TTG GTA AAC TAT ATC TTG TGT G	SacI
SP1294_Na_J4	CTC AAT TTT ATC TGA TGA TTG C	
SP0423-427_J1	TTCATCAAATGATGCGATT	
SP0423-427_J2	CA<u>**G GTA CC**</u>A ACG TTT GAA AGC AAG AAC T	KpnI
SP0423-427_J3	CA<u>**G AGC TC**</u>T GAG ACT AGA AAG GTC TCA TTT T	SacI
SP0423-427_J4	AAAGGTTGGTTGTCCATTT	
SP1855_adh_J1	GAG CGA TTG GAC AAT GTG G	
SP1855_adh_J2	CA<u>**G GTA CC**</u>C GAT TTA AAG CTG TCT GTA GCT GA	KpnI
SP1855_adh_J3	CA<u>**G AGC TC**</u>G GTC AGG CTA GGG ATT TTC C	SacI
SP1855_adh_J4	CTG AAG AGC TGG GCT ACA CC	
SP0783_bioY_J1	CGAGAAAGAACTGACTCCAG	
SP0783_bioY_J2	CA<u>**G GTA CC**</u>GAGTCGATGTCAACCATTTT	KpnI
SP0783_bioY_J3	CA<u>**G AGC TC**</u>CAAAGAGCAAACTAGGAAGC	SacI
SP0783_bioY_J4	CAGGTTGGTCATATCCATTT	
SP1270_orf3_J1	AGGTGATCGACCTATTTTGA	
SP1270_orf3_J2	CA<u>**G GTA CC**</u>ATGCATCTGTCAAGATTTCC	KpnI
SP1270_orf3_J3	CA<u>**G AGC TC**</u>GTACTGGAGGTTAATTGTGGA	SacI
SP1270_orf3_J4	TTTTCTCTAAGGAGAAGACTGC	
SP1922_hypo_J1	AATGTTTTAGAAGTGCCAGTGT	
SP1922_hypo_J2	CA<u>**G GTA CC**</u>AGGAAATGGATTTTATTAGCAA	KpnI
SP1922_hypo_J3	CA<u>**G AGC TC**</u>GTGCAGCCTTTGGTAAAA	SacI
SP1922_hypo_J4	TCTCCCCCTTCTAAATAAAAA	
SP0462-0468_rlr_J1	TCAAAAAGGTTGTGGAATTT	
SP0462-0468_rlr_J2	CA<u>**G GTA CC**</u>ATCGAAGAAAAAGCTGTCAA	KpnI
SP0462-0468_rlr_J3	CA<u>**G AGC TC**</u>GGACACAGAGATTATTTTACCGTCG	SacI
SP0462-0468_rlr_J4	TTTATCGCCCTACTTTTCGTATGC	

*The primer name is based on the concatenation of the gene locus ID, our own brief gene name and the Janus primer ID
**Restriction sites added to the primers are in bold and underlined
