# Supplementary material for: Phenotypic, genomic, and transcriptional characterization of Streptococcus pneumoniae interacting with human pharyngeal cells
Source: BMC Genomics. 2013 Jun 9;14:383. doi: 10.1186/1471-2164-14-383 (PMC3708772; doi:10.1186/1471-2164-14-383)
Supplement: Additional file 4 — Is a table listing the primers used for RT-PCR confirmation of the pneumolysin operon. [file 1471-2164-14-383-S4.pdf]

Additional data file 4. List of primers used for RT-PCR confirmation of the pneumolysin operon.

Primers used to make full-length cDNAs:

1920_F	GCCTGTAGTTGACCGAGAACC
1922_F	GCCTTGTGAAGGTCAGTTGG
1923_F	GCTGTAAAGCGACTGCCTTC
1924_F	GACTTGTTCAGCGAAATCAGC
1926_F	GCAAGCCGAGACTGGATAAG
1922_R	ATCGACCGTGCTAAACAAGC
1923_R	AGCGGTAAACGATTTGTTGG
1923_R2	GAAATGGGCAGGATTTAACG
1924_R	GGCTGATTTCGCTGAACAAG
1926_R	CTTATCCAGTCTCGGCTTGC

Primers used for PCR on cDNAs:

1922_F	GCCTTGTGAAGGTCAGTTGG
1923_F	GCTGTAAAGCGACTGCCTTC
1924_F	GACTTGTTCAGCGAAATCAGC
1926_F	GCAAGCCGAGACTGGATAAG
1922_R	ATCGACCGTGCTAAACAAGC
1923_R	AGCGGTAAACGATTTGTTGG
1923_R2	GAAATGGGCAGGATTTAACG
1924_R	GGCTGATTTCGCTGAACAAG
1926_R	CTTATCCAGTCTCGGCTTGC
1922new_F	TGATTCTGGATCTGGATCACC
1923new_R	ATGGTGGCGTACGGTTTATG

**Loci spanned**	**F-Primer**	**R-Primer**	**Prod Size**	**GEL lanes**
SP_1922–SP_1923	1922_F	1923_R2	1640	2, 3
SP_1923–SP_1924	1923_F	1924_R	1031	4, 5
SP_1924–SP_1926	1924_F	1926_R	1261	6, 7
SP_1922–SP_1923	1922new_F	1923new_R	1123	8, 9
SP_1922 (intragenic)	1922_F	1922_R	389	10, 11
SP_1923 (intragenic)	1923_F	1923_R	591	12, 13
SP_1926–SP_1927	1926_F	1927_R	682	14, 15
