# Supplementary material for: Phenotypic, genomic, and transcriptional characterization of Streptococcus pneumoniae interacting with human pharyngeal cells
Source: BMC Genomics. 2013 Jun 9;14:383. doi: 10.1186/1471-2164-14-383 (PMC3708772; doi:10.1186/1471-2164-14-383)

Additional data file 5. G54 wildtype, TIGR4 wildtype and isogenic mutant growth in EMEM without L-glutamine and supplemented with 7% fetabl bovine serum. At predetermined time points over a 3-h incubation period, culture was plated for enumeration. The error bars indicate SD between three replicate experiments. A) Growth of wildtype strains G54 and TIGR4Z5. P value as determined by t-test at 120 min; B) Growth of wild type strain TIGR4Z5 and its isogenic mutants.

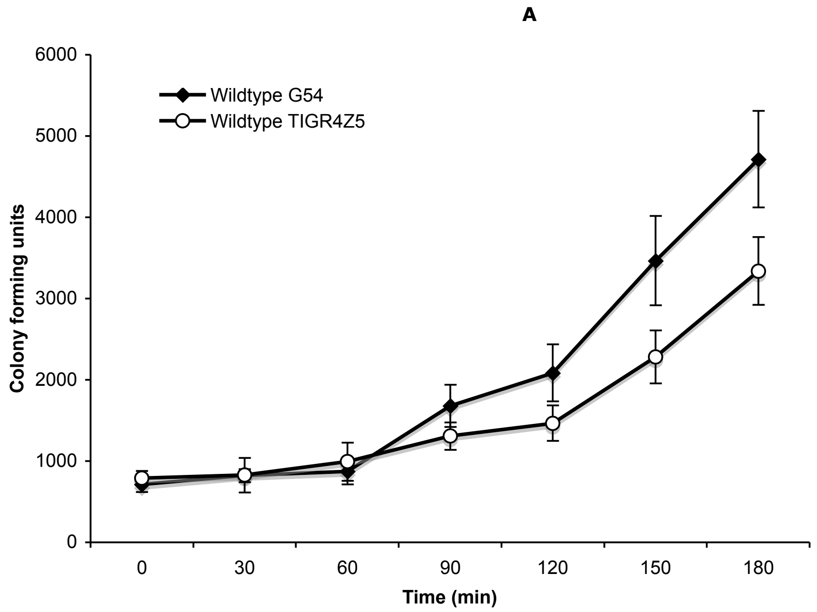

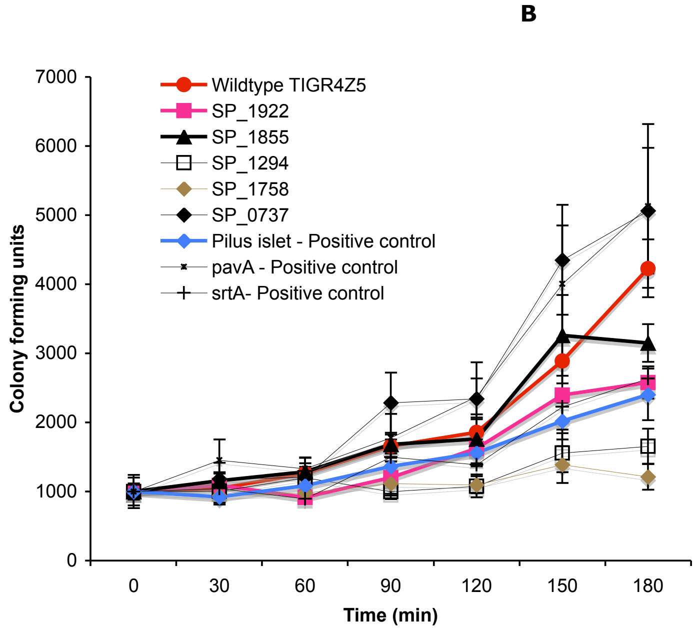

Supplement: Additional file 5 — Shows growth curves in EMEM of G54 and TIGR4 wild type strains, as well as the TIGR4 isogenic mutants. [file 1471-2164-14-383-S5.pdf]
