# Supplementary material for: Phenotypic, genomic, and transcriptional characterization of Streptococcus pneumoniae interacting with human pharyngeal cells
Source: BMC Genomics. 2013 Jun 9;14:383. doi: 10.1186/1471-2164-14-383 (PMC3708772; doi:10.1186/1471-2164-14-383)
Supplement: Additional file 6 — Is a table listing the regions of diversity between TIGR4 and G54 strain. [file 1471-2164-14-383-S6.pdf]

Additional data file 6. Regions of diversity (RD) and other strain-specific clusters present in *S. pneumoniae* strains TIGR4 and G54.

RD*	Genes in RD	Genes included	TIGR4	G54
1	SP_0067-74	Zinc metalloproteinase C (*zmpC*)	Present	Present
2	SP_0163-171	Macrolide efflux	Present	Absent
3	SP_0346-360	Capsular biosynthesis	Present	Present
4	SP_0461-468	*rlr* pathogenicity islet	Present	Absent
5	SP_0691-700	Hypothetical proteins	Present	Absent
6	SP_1028-1065	PPII, pneumococcal pathogenicity island I	Present	20/32 genes present
7	SP_1129-1147	Phage-related island (xer-type)	Present	3/11 genes present
8	SP_1315-1351	V-type sodium ATP synthase	Present	Present
9	SP_1612-1623	PTS system	Present	3/9 genes present
10	SP_1755-1773	*psrP-secY2A2* pathogenicity island	Present	Absent
11	SP_1828-1831	Phosphate transport system (*PhoU*)	Present	1/4 genes present
12	SP_1948-1955	Cytolysin	Present	3/9 present
13	SP_2168-2498	PTS system	Present	2/9 present
Other clusters:				
NA	SP_0109-115	Bacteriocin/ABC transporter	Present	Absent
NA	SP_0302-310	PTS system	Present	Absent
NA	SP_0381-387	Mevalonate pathway	Present	Absent
NA	SP_1433-1438	ABC transporter	Present	Absent
NA	SP_1796-1798	ABC transporter	Present	Absent
NA	SPG_0112-5,117	Conserved hypothetical protein	Absent	Present
NA	SPG_0225-227	ABC transporter, permease protein	Absent	Present
NA	SPG_0976-986	MutR, ABC transporter, permease protein	Absent	Present
NA	SPG_1231-1286	ermAm and tetM resistance genes	Absent	Present
NA	SPG_1856-1857	Hypothetical proteins	Absent	Present
NA	SPG_2099-2107	ABC transporter, glycosyl hydrolase, hypothetical prote	Absent	Present

*Tettelin *et al. [1]* carried out a genome comparison of 13 pneumococcal strains using comparative genome hybridization. TIGR4 was used as the reference strain. They identified a gene set consisting of 13 regions that were not part of the core genome (accessory genome) and these are referred to as regions of diversity (RD).

1. Tettelin H, Hollingshead S: **Comparative genomics of Streptococcus pneumoniae: intrastrain diversity and genome plasticity**. *The pneumococcus* 2004:p. 15–29.
