# Supplementary material for: Phenotypic, genomic, and transcriptional characterization of Streptococcus pneumoniae interacting with human pharyngeal cells
Source: BMC Genomics. 2013 Jun 9;14:383. doi: 10.1186/1471-2164-14-383 (PMC3708772; doi:10.1186/1471-2164-14-383)
Supplement: Additional file 7 — Is a table listing genes differentially expressed in cell-adherent pneumococci vs. culture medium control pneumococci. [file 1471-2164-14-383-S7.pdf]

Additional data file 7. List of genes differentially expressed in pharyngeal cell-adherent pneumococci vs. culture medium control pneumococci. Numbers in bold type represent genes that met the criteria of being both statistically differentially expressed and having a ratio of ≥ 2 or ≤ 0.5 in both strains. This gene list was supplemented with genes that did not meet the fold threshold value but appeared to be co-regulated as part of an operon. NA indicates data points that were removed after analysis with Spotfinder. X indicates genes that did not meet the criteria established for microarray analysis in MeV (see methods).

Locus	Common Name	Gene Symbol	Fold increase or decrease		Function
			TIGR4 adherent/ control	G54 adherent/ control	
SP_0014	Transcriptional regulator ComX1	*comX1*	X	2.9	Cellular processes/pathogenesis/Regulatory function
SP_0018	Hypothetical protein		1.7	3.3	Hypothetical proteins
SP_0019	Adenylosuccinate synthetase	*purA*	1.0	1.7	Purines, pyrimidines, nucleosides, and nucleotides
SP_0020	Cytidine-deoxycytidylate deaminase family protein		1.3	2.5	Unknown protein
SP_0024	Conserved hypothetical protein		2.6	0.9	Hypothetical proteins
SP_0025	Hypothetical protein		2.7	1.1	Hypothetical proteins
SP_0026	Hypothetical protein		2.2	X	Hypothetical proteins
SP_0042	Transport atp-binding protein ComA	*comA*	X	2.4	Cellular processes/pathogenesis/Transport and binding proteins
SP_0043	Transport protein	*comB*	X	2.6	Cellular processes/pathogenesis/Transport and binding proteins
SP_0044	Phosphoribosylaminoimidazole-succinocarboxamide synthase	*purC*	0.2	0.6	Cellular processes/pathogenesis
SP_0045	Phosphoribosylformylglycinamidine synthase II	*purL*	**0.2**	**0.4**	Cellular processes/pathogenesis/Purines, pyrimidines, nucleosides,
SP_0046	Amidophosphoribosyltransferase		**0.4**	**0.4**	Purines, pyrimidines, nucleosides, and nucleotides
SP_0047	Phosphoribosylformylglycinamide cyclo-ligase		**0.4**	**0.5**	Purines, pyrimidines, nucleosides, and nucleotides
SP_0048	Phosphoribosylglycinamide formyltransferase		**0.5**	**0.4**	Purines, pyrimidines, nucleosides, and nucleotides
SP_0049	Vanz protein, putative		0.6	0.5	Unknown protein
SP_0050	Phosphoribosylaminoimidazolecarboxamide formyltransferase-imp cyclohydrolase	*purH*	**0.5**	**0.4**	Purines, pyrimidines, nucleosides, and nucleotides
SP_0051	Phosphoribosylamine--glycine ligase		**0.3**	**0.4**	Purines, pyrimidines, nucleosides, and nucleotides
SP_0052	Hypothetical protein		**0.3**	**0.4**	Hypothetical proteins
SP_0053	Phosphoribosylaminoimidazole carboxylase, catalytic subunit	*purE*	**0.4**	**0.5**	Cellular processes/pathogenesis/Purines, pyrimidines, nucleosides,
SP_0054	Phosphoribosylaminoimidazole carboxylase, ATPase subunit		**0.4**	**0.4**	Cellular processes/pathogenesis/Purines, pyrimidines, nucleosides,
SP_0071	Zinc metalloprotease ZmpC	*zmpC*	0.6	0.4	Cellular processes/pathogenesis/Protein fate
SP_0083	DNA-binding response regulator		0.5	1.0	Signal transduction
SP_0084	Sensor histidine kinase		0.4	0.8	Signal transduction
SP_0099	Hypothetical protein		2.2	1.2	Hypothetical proteins
SP_0100	Conserved hypothetical protein		2.2	1.4	Hypothetical proteins
SP_0101	Putative transporter		1.4	1.9	Transport and binding proteins
SP_0103	Putative capsular polysaccharide biosynthesis protein		1.6	2.4	Cell envelope
SP_0107	LysM domain protein		1.4	2.7	Cell envelope
SP_0110	Bacteriocin-associated integral membrane protein		0.8	0.6	Cellular processes/pathogenesis
SP_0111	Putative amino acid ABC transporter, ATP-binding protein		1.1	0.6	Transport and binding proteins
SP_0115	Hypothetical protein		0.8	0.7	Hypothetical proteins
SP_0124	Hypothetical protein		X	3.3	Hypothetical proteins
SP_0126	Hypothetical protein		X	1.9	Hypothetical proteins
SP_0139	Conserved domain protein		X	0.4	Hypothetical proteins
SP_0140	Udp-glucose 6-dehydrogenase, authentic frameshift	*ugd*	X	0.4	Cell envelope
SP_0142	Hypothetical protein		1.3	0.7	Hypothetical proteins
SP_0143	Conserved domain protein		1.3	0.6	Hypothetical proteins
SP_0144	Hypothetical protein		1.8	0.6	Hypothetical proteins
SP_0145	Conserved hypothetical protein		1.6	0.7	Hypothetical proteins
SP_0146	Conserved hypothetical protein		1.3	0.7	Hypothetical proteins
SP_0147	Hypothetical protein		1.4	0.6	Hypothetical proteins
SP_0148	ABC transporter, substrate-binding protein		0.4	0.7	Transport and binding proteins
SP_0149	Lipoprotein		X	0.6	Cell envelope
SP_0158	Nrdi family protein		1.8	1.5	Unknown protein
SP_0159	Conserved hypothetical protein		0.7	0.3	Hypothetical proteins
SP_0169	Lactose phosphotransferase system repressor, degenerate		2.1	NA	Disrupted reading frame
SP_0170	Hypothetical protein		2.1	NA	Hypothetical proteins
SP_0181	Conserved hypothetical protein		2.3	1.3	Hypothetical proteins
SP_0267	N5,n10-methylenetetrahydromethanopterin reductase homolog.		0.3	X	Unknown protein
SP_0282	PTS system, mannose-specific IID component		1.0	0.5	Transport and binding proteins/Signal transduction
SP_0283	PTS system, mannose-specific IIC component	*manM*	0.8	0.5	Transport and binding proteins/Signal transduction
SP_0284	PTS system, mannose-specific IIAB components	*manL*	0.8	0.3	Transport and binding proteins/Signal transduction
SP_0285	Alcohol dehydrogenase, propanol-preferring		**0.4**	**0.3**	Energy metabolism
SP_0287	Xanthine-uracil permease family protein		**0.2**	**0.4**	Transport and binding proteins
SP_0288	Conserved hypothetical protein		**0.4**	**0.4**	Hypothetical proteins
SP_0289	Dihydropteroate synthase		0.6	0.6	Biosynthesis of cofactors, prosthetic groups, and carriers/ Cellular processes/pathogenesis/Energy metabolism
SP_0290	Dihydrofolate synthetase.		0.7	0.5	Biosynthesis of cofactors, prosthetic groups, and carriers
SP_0291	GTP cyclohydrolase I		0.7	0.6	Biosynthesis of cofactors, prosthetic groups, and carriers
SP_0292	Bifunctional folate synthesis protein		0.6	0.5	Biosynthesis of cofactors, prosthetic groups, and carriers
SP_0366	Oligopeptide-binding protein alia precursor (exported protein 1)		1.0	0.3	Transport and binding proteins
SP_0371	Conserved hypothetical protein		2.0	X	Hypothetical proteins
SP_0375	6-phosphogluconate dehydrogenase, decarboxylating		0.5	0.6	Energy metabolism
SP_0409	Conserved hypothetical protein		0.6	0.4	Hypothetical proteins
SP_0415	Enoyl-coa hydratase	*phaB*	1.5	0.4	Fatty acid and phospholipid metabolism
SP_0416	Transcriptional regulator, MarR family	*marR*	1.7	X	Regulatory function
SP_0417	3-oxoacyl-(acyl-carrier-protein) synthase III		1.6	1.0	Fatty acid and phospholipid metabolism
SP_0418	Acyl carrier protein		1.8	0.9	Fatty acid and phospholipid metabolism
SP_0419	Enoyl-(acyl-carrier-protein) reductase	*fabK*	1.4	0.4	Fatty acid and phospholipid metabolism
SP_0420	Malonyl coa-acyl carrier protein transacylase		1.9	0.6	Fatty acid and phospholipid metabolism
SP_0421	3-oxoacyl-[acyl-carrier protein] reductase		2.7	0.7	Fatty acid and phospholipid metabolism
SP_0422	3-oxoacyl-(acyl-carrier-protein) synthase II	*fabF*	2.4	0.7	Fatty acid and phospholipid metabolism
SP_0423	Acetyl-CoA carboxylase, bitoin carboxyl carrier protein		3.6	0.9	Fatty acid and phospholipid metabolism
SP_0424	Similar to hydroxymyristoyl-(acyl carrier protein) dehydratase.		3.6	1.0	Fatty acid and phospholipid metabolism
SP_0425	Acetyl-coa carboxylase, biotin carboxylase		4.2	1.1	Fatty acid and phospholipid metabolism
SP_0426	Acetyl-coa carboxylase, carboxyl transferase subunit beta	*accD*	5.5	1.4	Fatty acid and phospholipid metabolism
SP_0427	Acetyl-coa carboxylase, carboxyl transferase subunit alpha	*accA*	2.5	1.6	Fatty acid and phospholipid metabolism
SP_0429	Hypothetical protein		X	2.8	Hypothetical proteins
SP_0430	Hypothetical protein		X	4.1	Hypothetical proteins
SP_0445	Acetolactate synthase, large subunit, biosynthetic type	*ilvB*	0.8	0.4	Amino acid biosynthesis
SP_0446	Acetolactate synthase, small subunit	*ilvN*	0.6	0.2	Amino acid biosynthesis
SP_0447	Ketol-acid reductoisomerase	*ilvC*	0.7	0.2	Amino acid biosynthesis
SP_0448	Hypothetical protein		0.8	0.2	Hypothetical proteins
SP_0449	Hypothetical protein		0.8	0.2	Hypothetical proteins
SP_0450	Threonine dehydratase	*ilvA*	1.0	0.4	Amino acid biosynthesis
SP_0461	Transcriptional regulator, putative		2.1	NA	Cellular processes/pathogenesis/Regulatory function
SP_0462	Cell wall surface anchor family protein		2.6	NA	Cell envelope/Cellular processes/pathogenesis
SP_0463	Cell wall surface anchor family protein		1.9	NA	Cell envelope/Cellular processes/pathogenesis
SP_0464	Cell wall surface anchor family protein		2.1	NA	Cell envelope/Cellular processes/pathogenesis
SP_0466	Sortase, putative		2.0	NA	Cell envelope/Cellular processes/pathogenesis/Protein fate
SP_0467	Sortase, putative		2.3	NA	Cell envelope/Cellular processes/pathogenesis/Protein fate
SP_0468	Sortase, putative		2.2	NA	Cell envelope/Cellular processes/pathogenesis/Protein fate
SP_0494	Ctp synthetase.		1.2	2.2	Purines, pyrimidines, nucleosides, and nucleotides
SP_0496	Na/Pi cotransporter II-related protein		1.3	1.7	Unknown protein

			Fold increase or decrease		
Locus	Common Name	Gene Symbol	TIGR4 adherent/ control	G54 adherent/ control	Function
SP_0528	Peptide pheromone BlpC	*blpC*	X	0.5	Cellular processes/pathogenesis/Transcription
SP_0529	Transport protein BlpB	*blpB*	X	0.4	Cellular processes/pathogenesis/Transport and binding proteins
SP_0530	Transport ATP-binding protein ComA	*comA*	X	0.4	Disrupted reading frame
SP_0535	Hypothetical protein		X	0.8	Hypothetical proteins
SP_0539	Bacteriocin BlpM	*blpM*	X	0.5	Cellular processes/pathogenesis
SP_0540	BlpN protein	*blpN*	X	0.5	Cellular processes/pathogenesis
SP_0541	Bacteriocin BlpO	*blpO*	1.1	0.6	Cellular processes/pathogenesis
SP_0543	Hypothetical protein		X	0.6	Hypothetical proteins
SP_0544	Immunity protein BlpX	*blpX*	X	0.6	Cellular processes/pathogenesis
SP_0545	Immunity protein BlpY	*blpY*	X	0.7	Cellular processes/pathogenesis
SP_0607	Amino acid abc transporter, permease protein		X	2.5	Transport and binding proteins
SP_0608	ABC transporter membrane-spanning permease	*glnP*	1.4	1.7	Transport and binding proteins
SP_0609	Amino acid ABC transporter, amino acid-binding protein		X	2.0	Cellular processes/pathogenesis/Transport and binding proteins
SP_0610	Amino acid ABC transporter, ATP-binding protein		1.3	1.8	Transport and binding proteins
SP_0617	Conserved domain protein		1.5	3.6	Hypothetical proteins
SP_0626	Branched-chain amino acid transport system II carrier protein	*brnQ*	1.0	2.1	Transport and binding proteins
SP_0685	Hypothetical protein		X	0.5	Hypothetical proteins
SP_0686	Bacteriocin-associated integral membrane protein		X	0.4	Cellular processes/pathogenesis
SP_0696	Hypothetical protein		2.1	NA	Hypothetical proteins
SP_0697	Abc transporter, atp-binding protein, authentic point mutation		1.8	NA	Transport and binding proteins
SP_0701	Orotidine 5'-phosphate decarboxylase		1.0	2.2	Purines, pyrimidines, nucleosides, and nucleotides
SP_0702	Orotate phosphoribosyltransferase PyrE		X	2.0	Purines, pyrimidines, nucleosides, and nucleotides
SP_0726	Phosphomethylpyrimidine kinase		0.3	1.1	Biosynthesis of cofactors, prosthetic groups, and carriers
SP_0730	Pyruvate oxidase		0.3	0.6	Cellular processes/pathogenesis/Energy metabolism
SP_0737	Sodium-dependent transporter		2.4	X	Cellular processes/pathogenesis/Transport and binding proteins
SP_0738	Conserved domain protein		2.0	X	Hypothetical proteins
SP_0753	Branched-chain amino acid ABC transporter, ATP-binding protein.		1.3	0.5	Transport and binding proteins
SP_0766	Manganese co-factored superoxide dismutase.		**0.3**	**0.5**	Cellular processes/pathogenesis
SP_0784	Glutathione reductase		**0.1**	**0.4**	Biosynthesis of cofactors, prosthetic groups, and carriers/Cellular pro
SP_0786	ABC transporter ATP-binding protein - unknown substrate		0.8	2.9	Transport and binding proteins
SP_0798	Dna-binding response regulator ciar	*ciaR*	1.2	2.9	Regulatory function/Signal transduction
SP_0799	Sensor histidine kinase CiaH	*ciaH*	1.3	3.1	Regulatory function/Signal transduction
SP_0820	ATP-dependent Clp protease, ATP-binding subunit ClpE	*clpE*	0.4	0.9	Cellular processes/pathogenesis/Protein fate
SP_0845	Lipoprotein		**0.5**	**0.4**	Cell envelope
SP_0856	Branched-chain amino acid aminotransferase	*ilvE*	1.3	0.5	Amino acid biosynthesis
SP_0857	Oligopeptide-binding protein, internal deletion, authentic point mutation		1.3	0.5	Disrupted reading frame
SP_0858	Membrane protein		1.3	0.5	Cell envelope
SP_0860	Pyrrolidone-carboxylate peptidase		1.4	0.5	Protein fate
SP_0867	ABC transporter, ATP-binding protein		0.3	0.7	Transport and binding proteins
SP_0868	Conserved hypothetical protein		**0.3**	**0.5**	Hypothetical proteins
SP_0869	YurW protein		**0.3**	**0.5**	Unknown protein
SP_0870	NifU family protein		**0.3**	**0.5**	Unknown protein
SP_0879	Hypothetical protein		**2.0**	**3.9**	Hypothetical proteins
SP_0999	Cytochrome c-type biogenesis protein CcdA	*ccdA*	0.3	0.6	Energy metabolism
SP_1000	Thioredoxin family protein		**0.2**	**0.4**	Energy metabolism
SP_1003	Conserved hypothetical protein	*phtD*	0.8	3.9	Hypothetical proteins
SP_1004	Hypothetical protein.		X	2.0	Hypothetical proteins
SP_1014	Dihydrodipicolinate synthase	*dapA*	1.0	0.4	Amino acid biosynthesis
SP_1027	Conserved hypothetical protein		1.7	6.0	Hypothetical proteins
SP_1045	Conserved hypothetical protein		0.4	0.9	Hypothetical proteins
SP_1128	Phosphopyruvate hydratase	*eno*	0.4	0.8	Energy metabolism
SP_1154	Immunoglobulin A1 protease		0.5	NA	Cellular processes/pathogenesis/Protein fate
SP_1174	Conserved domain protein		X	3.3	Hypothetical proteins
SP_1175	Conserved domain protein		1.1	2.9	Hypothetical proteins
SP_1226	Histidine kinase		0.5	0.9	Signal transduction
SP_1227	DNA-binding response regulator		0.5	0.9	Signal transduction
SP_1228	YfhQ protein		0.3	0.9	DNA metabolism
SP_1229	Formate--tetrahydrofolate ligase		0.3	0.9	Central intermediary metabolism
SP_1230	Conserved hypothetical protein		0.5	1.2	Hypothetical proteins
SP_1231	Flavoprotein		0.5	1.3	Unknown protein
SP_1241	Amino acid ABC transporter, amino acid-binding protein/permease protein		1.6	2.7	Transport and binding proteins
SP_1242	Amino acid ABC transporter, ATP-binding protein		0.5	2.7	Transport and binding proteins
SP_1252	Hypothetical protein		X	1.5	Hypothetical proteins
SP_1253	Hypothetical protein		X	1.9	Hypothetical proteins
SP_1254	Hypothetical protein		X	1.9	Hypothetical proteins
SP_1255	Putative 3-isopropylmalate dehydratase, small subunit		X	2.1	Amino acid biosynthesis
SP_1256	Conserved hypothetical protein		X	1.9	Hypothetical proteins
SP_1257	3-isopropylmalate dehydrogenase, authentic point mutation	*leuB*	X	1.4	Amino acid biosynthesis
SP_1258	Putative 2-isopropylmalate synthase		X	1.9	Amino acid biosynthesis
SP_1259	Conserved hypothetical protein		X	1.7	Hypothetical proteins
SP_1260	Copper homeostasis protein, CutC	*cutC*	1.2	1.8	Transport and binding proteins
SP_1261	Conserved hypothetical protein		1.5	2.3	Hypothetical proteins
SP_1266	DNA processing protein DprA, putative		X	3.6	Cellular processes/pathogenesis
SP_1267	LicC protein	*licC*	**4.0**	**3.4**	Cell envelope
SP_1268	LicB	*licB*	**4.0**	**3.2**	Cell envelope
SP_1269	Choline kinase.		**3.1**	**3.6**	Cell envelope
SP_1270	Alcohol dehydrogenase, zinc-containing		**3.1**	**3.6**	Cell envelope/Energy metabolism
SP_1271	Putative 2-C-methyl-D-erythritol 4-phosphate cytidylyltransferase		**2.1**	**2.9**	Biosynthesis of cofactors, prosthetic groups, and carriers
SP_1272	Repeating unit transporter.		1.6	1.9	Cell envelope
SP_1273	LicD1	*licD1*	1.3	1.7	Cell envelope
SP_1274	LicD2protein	*licD2*	1.4	2.0	Cell envelope
SP_1275	Carbamoyl-phosphate synthase, large subunit	*carB*	0.5	1.4	Purines, pyrimidines, nucleosides, and nucleotides
SP_1276	Carbamoyl-phosphate synthase, small subunit		0.6	1.1	Purines, pyrimidines, nucleosides, and nucleotides
SP_1277	Aspartate carbamoyltransferase		0.5	1.1	Purines, pyrimidines, nucleosides, and nucleotides
SP_1294	CrcB protein	*crcB*	2.0	1.1	Unknown protein
SP_1295	CrcB protein	*crcB*	1.8	1.1	Unknown protein
SP_1296	Putative chorismate mutase		1.9	1.2	Amino acid biosynthesis
SP_1357	ABC transporter, permease/ATP-binding protein		0.9	0.4	Transport and binding proteins
SP_1358	ABC transporter, ATP-binding protein.		0.7	0.3	Transport and binding proteins
SP_1359	Peptide methionine sulfoxide reductase		0.7	0.5	Cell envelope/Cellular processes/pathogenesis/Protein fate
SP_1360	Homoserine kinase		**0.5**	**0.5**	Amino acid biosynthesis
SP_1361	Homoserine dehydrogenase		0.6	0.4	Amino acid biosynthesis
SP_1429	Peptidase, U32 family		**2.1**	**2.5**	Protein fate
SP_1460	Probable amino-acid ATP transporter ATP-binding protein YckI.		0.6	0.6	Transport and binding proteins
SP_1461	Amino acid ABC transporter, permease protein		0.6	0.6	Transport and binding proteins
SP_1463	Methylated-dna--protein-cysteine s- methyltransferase		0.6	0.6	DNA metabolism
SP_1464	Acetyltransferase, GNAT family		**0.5**	**0.5**	Unknown protein

Locus	Common Name	Gene Symbol	Fold increase or decrease: TIGR4 adherent/ control	Fold increase or decrease: G54 adherent/ control	Function
SP_1465	Hypothetical protein		0.7	0.3	Hypothetical proteins
SP_1499	Bacterocin transport accessory protein		**0.2**	**0.5**	Cellular processes/pathogenesis/Transport and binding proteins
SP_1546	Hypothetical protein		**0.1**	**0.4**	Hypothetical proteins
SP_1550	Glutathione S-transferase family protein		1.1	0.3	Central intermediary metabolism
SP_1551	P-type ATPase - calcium transporter	*pacL*	0.9	0.3	Transport and binding proteins
SP_1572	Surface located protein.		1.1	2.2	Transport and binding proteins
SP_1586	Autoaggregation-mediating protein		**0.4**	**0.4**	Transcription
SP_1587	Oxalate-formate antiporter (OxlT-2).		**0.2**	**0.2**	Transport and binding proteins
SP_1588	Oxidoreductase, pyridine nucleotide-disulfide, class I		**0.1**	**0.2**	Unknown protein
SP_1596	Is3-spn1, hypothetical protein, interruption		1.0	0.5	Disrupted reading frame
SP_1600	Putative membrane protein		**2.0**	**2.0**	Hypothetical proteins
SP_1601	Conserved hypothetical protein		**2.0**	**2.0**	Hypothetical proteins
SP_1602	Required for expression of the phosphonate utilization phenotype in *E. coli*	*phnA*	**2.5**	**2.1**	Transport and binding proteins
SP_1648	Manganese ABC transporter, ATP-binding protein	*psaB*	**2.8**	**2.8**	Transport and binding proteins
SP_1649	Manganese ABC transporter, permease protein, putative, authentic frameshift	*psaC*	**2.4**	**2.8**	Transport and binding proteins
SP_1650	Manganese abc transporter, manganese-binding adhesion liprotein		**2.0**	**2.5**	Cellular processes/pathogenesis/Transport and binding proteins
SP_1651	Thiol peroxidase		**0.3**	**0.3**	Cellular processes/pathogenesis
SP_1653	ABC transporter, ATP-binding protein.		0.9	0.5	Transport and binding proteins
SP_1654	Conserved hypothetical protein		X	0.3	Hypothetical proteins
SP_1695	Xylan esterase 1		X	3.3	Energy metabolism
SP_1696	Hypothetical protein		1.1	2.5	Hypothetical proteins
SP_1708	Hypothetical protein		2.2	0.8	Hypothetical proteins
SP_1714	transcriptional regulator, GntR family		1.4	2.0	Regulatory function
SP_1715	ABC transporter, ATP-binding protein		1.1	2.2	Transport and binding proteins
SP_1716	Conserved hypothetical protein		X	2.7	Hypothetical proteins
SP_1717	ABC transporter, ATP-binding protein		X	3.0	Transport and binding proteins
SP_1754	Conserved hypothetical protein		1.8	0.5	Hypothetical proteins
SP_1758	Glycosyl transferase, group 1		2.2	NA	Cell envelope
SP_1761	Hypothetical protein		1.9	NA	Hypothetical proteins
SP_1762	Hypothetical protein		2.1	NA	Hypothetical proteins
SP_1770	Glycosyl transferase, family 8		2.2	NA	Cell envelope
SP_1771	Glycosyl transferase, family 2-glycosyl transferase family 8		1.8	NA	Cell envelope
SP_1774	Transcriptional regulator, putative		**0.5**	**0.5**	Regulatory function
SP_1775	Hypothetical protein		**0.2**	**0.4**	Hypothetical proteins
SP_1776	Thioredoxin reductase	*trxA*	**0.2**	**0.3**	Energy metabolism
SP_1778	Water channel protein.		0.5	0.7	Transport and binding proteins
SP_1811	Tryptophan synthase, alpha subunit		1.9	0.7	Amino acid biosynthesis
SP_1813	N-(5'-phosphoribosyl)-anthranilate isomerase		**2.0**	**2.0**	Amino acid biosynthesis
SP_1814	Indole-3-glycerol phosphate synthase		1.8	1.8	Amino acid biosynthesis
SP_1845	Exodeoxyribonuclease		0.4	0.7	DNA metabolism
SP_1848	Xanthine permease		0.3	X	Transport and binding proteins
SP_1853	Galactokinase	*galK*	1.1	0.3	Energy metabolism
SP_1855	Dehydrogenase		6.0	1.0	Energy metabolism
SP_1856	Transcriptional regulator, MerR family		7.6	1.1	Regulatory function
SP_1857	Cation efflux system protein		14.4	X	Transport and binding proteins
SP_1860	Choline transporter	*proWX*	**0.2**	**0.5**	Transport and binding proteins
SP_1861	ABC transporter ATP-binding protein - choline transporter	*proV*	**0.2**	**0.5**	Transport and binding proteins
SP_1862	Hypothetical protein		**0.2**	**0.4**	Hypothetical proteins
SP_1872	Ferric anguibactin-binding protein precusor fatb of *V. anguillarum*		X	0.3	Transport and binding proteins
SP_1887	Oligopeptide transport ATP-binding protein AmiF	*amiF*	1.0	0.5	Transport and binding proteins
SP_1888	Oligopeptide ABC transporter, ATP-binding protein AmiE	*amiE*	1.0	0.5	Transport and binding proteins
SP_1889	Oligopeptide abc transporter, permease protein AmiD	*amiD*	1.0	0.5	Cellular processes/pathogenesis/Transport and binding proteins
SP_1890	Oligopeptide transport system permease protein AmiC	*amiC*	1.0	0.5	Transport and binding proteins
SP_1891	Oligopeptide-binding protein AmiA precursor.	*amiA*	0.8	0.5	Cellular processes/pathogenesis/Transport and binding proteins
SP_1893	Hypothetical protein		1.2	1.4	Hypothetical proteins
SP_1906	Chaperonin, 60 kDa		0.3	1.1	Protein fate
SP_1907	Chaperonin, 10 kDa		0.4	1.2	Protein fate
SP_1908	Single-strand binding protein (ssb) (helix-destabilizing protein).		X	3.5	Cellular processes/pathogenesis/DNA metabolism
SP_1922	Conserved hypothetical protein		2.3	1.0	Hypothetical proteins
SP_1923	Pneumolysin	*ply*	**3.0**	**0.5**	Cellular processes/pathogenesis
SP_1924	Hypothetical protein		**2.6**	**0.4**	Hypothetical proteins
SP_1925	Hypothetical protein		**2.3**	**0.4**	Hypothetical proteins
SP_1926	Hypothetical protein		1.7	0.4	Hypothetical proteins
SP_1945	Hypothetical protein		X	4.0	Hypothetical proteins
SP_1954	Serine protease, subtilase family, authentic frameshift		0.5	NA	Protein fate
SP_1986	Hypothetical protein		1.1	3.6	Hypothetical proteins
SP_1987	ABC transporter, ATP-binding protein		0.9	3.8	Transport and binding proteins
SP_1988	Bacteriocin-associated integral membrane protein		0.9	4.0	Cellular processes/pathogenesis
SP_2026	Alcohol-acetaldehyde dehydrogenase		X	0.4	Energy metabolism
SP_2044	Acetate kinase		0.6	0.5	Energy metabolism
SP_2053	Competence protein		X	3.3	Cellular processes/pathogenesis
SP_2054	Hypothetical protein		0.3	1.3	Hypothetical proteins
SP_2055	Alcohol dehydrogenase		**0.4**	**0.4**	Energy metabolism
SP_2108	Maltose-maltodextrin-binding protein precursor		0.7	0.4	Transport and binding proteins
SP_2109	Maltodextrin transport system permease protein MalC	*malC*	0.9	0.6	Transport and binding proteins
SP_2110	Maltodextrin ABC transporter, permease protein	*malD*	2.5	0.6	Transport and binding proteins
SP_2125	Conserved hypothetical protein		1.3	0.4	Hypothetical proteins
SP_2136	PcpA	*pcpA*	**0.5**	**2.4**	Cell envelope/Cellular processes/pathogenesis
SP_2169	Zinc ABC transporter, zinc-binding lipoprotein		1.3	1.8	Cellular processes/pathogenesis/Transport and binding proteins
SP_2170	Zinc ABC transporter, permease protein	*adcB*	1.5	2.8	Cellular processes/pathogenesis/Transport and binding proteins
SP_2171	Zinc abc transporter, atp-binding protein	*adcC*	1.4	2.6	Cellular processes/pathogenesis/Transport and binding proteins
SP_2172	Adc operon repressor AdcR	*adcR*	1.6	2.4	Cellular processes/pathogenesis/Regulatory function
SP_2173	Extramembranal protein		1.5	2.9	Cell envelope
SP_2174	D-alanyl carrier protein	*dltC*	1.7	2.7	Cell envelope
SP_2175	Integral membrane protein	*dltB*	1.3	2.4	Cell envelope/Transport and binding proteins
SP_2176	D-alanine-activating enzyme	*dltA*	1.4	2.5	Cell envelope
SP_2177	Hypothetical protein		1.2	2.3	Hypothetical proteins
SP_2187	Conserved domain protein		0.3	0.9	Hypothetical proteins
SP_2188	Chaperonin, 33 kDa		0.5	1.3	Protein fate
SP_2189	TIM-barrel protein, putative, NifR3 family		0.5	1.1	Unknown protein
SP_2197	ABC transporter, substrate-binding protein, putative		**0.4**	**0.4**	Transport and binding proteins
SP_2198	ABC transporter, permease protein		0.5	X	Transport and binding proteins
SP_2199	Conserved hypothetical protein		**0.4**	**0.5**	Hypothetical proteins
SP_2235	Response regulator come.	*comE*	X	2.8	Cellular processes/pathogenesis/Regulatory function/Signal transdu[illegible]
SP_2236	Putative sensor histidine kinase comd	*comD*	**2.3**	**3.1**	Cellular processes/pathogenesis/Regulatory function/Signal transdu[illegible]
SP_2237	Competence stimulating peptide precursor (CSP)	*comC*	1.3	3.1	Cellular processes/pathogenesis
SP_2239	HtrA protein	*htrA*	1.2	4.6	Protein fate
SP_2240	SpspoJ protein		1.4	4.3	Cellular processes/pathogenesis

* "NA" indicates data points that were removed after analysis with spotfinder. "X" indicates genes that do not meet the criteria established for microarray analysis in MeV (see experimental procedures)
