# Supplementary material for: Phenotypic, genomic, and transcriptional characterization of Streptococcus pneumoniae interacting with human pharyngeal cells
Source: BMC Genomics. 2013 Jun 9;14:383. doi: 10.1186/1471-2164-14-383 (PMC3708772; doi:10.1186/1471-2164-14-383)
Supplement: Additional file 8 — Is a table listing genes differentially expressed in cell-adherent pneumococci vs. non-adherent pneumococci exposed to D562 pharyngeal cells. [file 1471-2164-14-383-S8.pdf]

Additional data file 8. List of genes differentially expressed in nasopharyngeal cell-adherent pneumococci vs. non-adherent pneumococci. Ratios depicted are ratios of ratios known as “ratio index”, i.e. (adherent/control) / (non-adherent/control). The use of a common reference implies that the resultant ratio is equivalent to adherent/non-adherent. Numbers in bold type represent genes that met the criteria of being both statistically differentially expressed and above the ratio cutoff of ≥ 1.5 or ≤ 0.7. Genes with ratios above the cutoff in both TIGR4 and G54 are underlined. Numbers in regular font represent ratios that were statistically significant but did not meet the fold change cutoff. Numbers in italics and grey font indicates ratios that did not meet the criteria established for statistical significance (see experimental procedures). NA indicates data points that were removed after analysis with Spotfinder.

Locus	Common name	Gene Symbol	Ratio index TIGR4	G54
SP_0005	Peptidyl-trna hydrolase	*pth*	**0.6**	0.8
SP_0013	Cell division protein	*ftsH*	**0.7**	*1.1*
SP_0018	Hypothetical protein		**1.9**	*1.0*
SP_0019	Adenylosuccinate synthetase	*purA*	*1.0*	**0.6**
SP_0024	Conserved hypothetical protein		*1.0*	**0.7**
SP_0027	Ribose phosphate pyrophosphokinase		1.3	**1.6**
SP_0034	Hypothetical 36.9 kda protein in lysp-nfo intergenic region		**1.6**	**1.9**
SP_0037	Fatty acid/phospholipid synthesis protein	*plsX*	**0.7**	*1.0*
SP_0042	Transport ATP-binding protein (ComA)	*comA*	*0.9*	**0.7**
SP_0043	Transport protein	*comB*	**0.7**	*0.8*
SP_0044	Phosphoribosylaminoimidazole-succinocarboxamide synthase	*purC*	*0.8*	**0.7**
SP_0045	Phosphoribosylformylglycinamidine synthase II	*purL*	*0.8*	**0.7**
SP_0046	Amidophosphoribosyltransferase		*1.1*	**0.7**
SP_0047	Phosphoribosylformylglycinamide cyclo-ligase		1.4	**0.7**
SP_0048	Phosphoribosylglycinamide formyltransferase		*1.3*	**0.6**
SP_0049	VanZ protein, putative		**1.7**	**0.6**
SP_0050	Phosphoribosylaminoimidazolecarboxamide formyltransferase-imp cyclohydrolase	*purH*	**1.7**	**0.7**
SP_0051	Phosphoribosylamine--glycine ligase		*0.9*	**0.6**
SP_0052	Hypothetical protein		*0.9*	**0.6**
SP_0054	Phosphoribosylaminoimidazole carboxylase, atpase subunit		*1.0*	**0.6**
SP_0056	Adenylosuccinate lyase		*1.0*	**0.7**
SP_0060	Beta-galactosidase	*bga*	**0.1**	*1.0*
SP_0063	Phosphotransferase system sugar-specific EIID component	*PTS-EIID*	NA	**0.6**
SP_0064	PTS system, IIA component		NA	**0.6**
SP_0071	Zinc metalloprotease ZmpC	*zmpC*	**0.6**	**0.7**
SP_0074	Acetyltransferase, cyse-laca-lpxa-NODI family		*1.0*	**1.5**
SP_0075	Phosphorylase, Pnp-Udp family		*1.0*	**1.7**
SP_0077	Hypothetical protein		*1.1*	**1.6**
SP_0079	Potassium uptake protein, Trk family		*1.4*	**1.5**
SP_0083	DNA-binding response regulator		**0.6**	*1.0*
SP_0084	Sensor histidine kinase		**0.6**	*1.0*
SP_0090	Sugar-binding transport protein.		**0.4**	*0.5*
SP_0095	YbfQ protein		**0.4**	*0.8*
SP_0097	Hypothetical protein		0.8	**0.5**
SP_0098	Hypothetical protein		0.8	**0.5**
SP_0099	Hypothetical protein		*0.8*	**0.5**
SP_0100	Conserved hypothetical protein		*0.8*	**0.5**
SP_0101	Putative transporter		1.2	**1.6**
SP_0103	Putative capsular polysaccharide biosynthesis protein		1.4	**1.8**
SP_0105	L-serine dehydratase, iron-sulfur-dependent, alpha subunit	*sdhA*	**0.7**	*1.0*
SP_0106	L-serine dehydratase beta subunit	*sdaB*	*1.0*	**1.6**
SP_0107	Lysm domain protein		**1.5**	*1.2*
SP_0109	Bacteriocin, putative		**0.7**	**0.6**
SP_0110	Bacteriocin-associated integral membrane protein		*0.7*	**0.7**
SP_0111	Putative amino acid ABC transporter, ATP-binding protein		*1.1*	**0.7**
SP_0117	Pneumococcal surface protein A	*pspA*	**0.5**	NA
SP_0132	Transposase	*transposase_A*	*1.2*	**0.7**
SP_0139	Conserved domain protein		NA	**0.4**
SP_0140	Udp-glucose 6-dehydrogenase, authentic frameshift	*ugd*	NA	**0.4**
SP_0144	Hypothetical protein		*1.3*	**0.5**
SP_0145	Conserved hypothetical protein		*1.2*	**0.6**
SP_0146	Conserved hypothetical protein		*0.9*	**0.6**
SP_0147	Hypothetical protein		*0.9*	**0.5**
SP_0148	ABC transporter, substrate-binding protein		**0.6**	**0.7**
SP_0149	Lipoprotein		NA	**0.6**
SP_0153	Hypothetical protein		**1.7**	NA
SP_0158	Nrdi family protein		**1.8**	**1.5**
SP_0159	Conserved hypothetical protein		1.3	**0.7**
SP_0175	6,7-dimethyl-8-ribityllumazine synthase	*ribE*	**0.6**	*0.8*
SP_0176	3,4-dihydroxy-2-butanone 4-phosphate synthase/ GTP cyclohydrolase II		**0.6**	NA
SP_0177	Riboflavin synthase subunit alpha	*ribC*	**0.7**	NA
SP_0190	Hypothetical protein		**0.7**	**0.7**
SP_0191	Hypothetical protein		**0.7**	**0.7**
SP_0194	Hypothetical protein		*1.0*	**0.7**
SP_0195	Hypothetical protein		0.8	**0.7**
SP_0196	Hypothetical protein		0.8	**0.7**
SP_0197	Putative dihydrofolate synthetase		**0.7**	0.8
SP_0202	Anaerobic ribonucleoside-triphosphate reductase	*nrdD*	**0.7**	*1.1*
SP_0205	Anaerobic ribonucleoside-triphosphate reductase activating protein	*nrdG*	**0.7**	NA
SP_0214	50S ribosomal protein L22	*rplV*	**0.7**	NA
SP_0218	Ribosomal protein S17	*rpsQ*	**0.7**	*0.9*
SP_0222	30s ribosomal protein s14 pseudogene.		**0.7**	*1.0*
SP_0267	N5,n10-methylenetetrahydromethanopterin reductase homolog.		**0.6**	*0.8*
SP_0281	Aminopeptidase c		*1.0*	**1.7**
SP_0282	PTS system, mannose-specific IID component		*1.3*	**0.6**
SP_0283	PTS system, mannose-specific IIC component	*manM*	**0.7**	**0.6**
SP_0284	PTS system, mannose-specific IIAB components	*manL*	**0.6**	**0.5**
SP_0285	Alcohol dehydrogenase, propanol-preferring		**0.4**	**0.3**
SP_0287	OrfX protein		*1.1*	**0.4**
SP_0288	Hypothetical protein		*1.0*	**0.5**
SP_0289	Dihydropteroate synthase		*0.8*	**0.6**
SP_0290	Dihydrofolate synthetase.		*0.9*	**0.5**
SP_0291	GTP cyclohydrolase		*1.0*	**0.7**
SP_0292	Aldolase-pyrophosphokinase	*sulD*	*1.0*	**0.5**
SP_0332	Hypothetical protein		*0.8*	**1.5**
SP_0333	Putative transcriptional regulator		*0.9*	**1.6**
SP_0337	Phospho-N-acetylmuramoyl-pentapeptide-transferase		NA	**1.5**
SP_0341	Hypothetical protein		*1.1*	**1.5**
SP_0342	Glucan 1,6-alpha-glucosidase	*dexB*	**0.5**	NA
SP_0366	Oligopeptide-binding protein alia precursor (exported protein 1).		**1.5**	**0.5**
SP_0375	6-phosphogluconate dehydrogenase, decarboxylating		**0.7**	*0.9*
SP_0390	Choline binding protein G	*cbpG*	**0.7**	*0.9*
SP_0410	Hypothetical protein		1.4	**1.5**
SP_0418	Acyl carrier protein	*acpP*	**1.6**	*0.8*
SP_0419	Enoyl-(acyl-carrier-protein) reductase	*fabK*	**0.5**	**0.6**
SP_0423	Acetyl-CoA carboxylase, bitoin carboxyl carrier protein		**2.1**	*1.8*

Locus	Common name	Gene Symbol	Ratio index	
			TIGR4	G54
SP_0424	Similar to hydroxymyristoyl-(acyl carrier protein) dehydratase.		**2.1**	*1.9*
SP_0425	Acetyl-coa carboxylase, biotin carboxylase		**2.1**	*2.1*
SP_0426	Acetyl-coa carboxylase, carboxyl transferase subunit beta	*accD*	**2.7**	*2.8*
SP_0427	Acetyl-coa carboxylase, carboxyl transferase subunit alpha	*accA*	**2.0**	*2.8*
SP_0437	Glutamyl-trna(Gln) amidotransferase, A subunit	*gatA*	**0.7**	1.3
SP_0441	Ribosomal protein L28	*rpmB*	**1.5**	*1.0*
SP_0445	Acetolactate synthase, large subunit, biosynthetic type		*1.3*	**0.5**
SP_0446	Acetolactate synthase, small subunit		*0.9*	**0.5**
SP_0447	Ketol-acid reductoisomerase		*0.9*	**0.4**
SP_0448	Hypothetical protein		*1.1*	**0.5**
SP_0449	Hypothetical protein		1.3	**0.5**
SP_0450	Threonine dehydratase	*ilvA*	1.3	**0.6**
SP_0459	Pyruvate formate-lyase		**0.3**	*0.9*
SP_0461	Transcriptional regulator, putative		**2.0**	NA
SP_0464	Cell wall surface anchor family protein		**2.0**	NA
SP_0465	Hypothetical protein		**1.6**	NA
SP_0466	Sortase, putative		**1.7**	NA
SP_0467	Sortase, putative		**1.9**	NA
SP_0468	Sortase, putative		**1.9**	NA
SP_0479	Trk potassium uptake system protein	*trkH*	NA	**1.5**
SP_0480	Trka protein (fragment).		*1.0*	**1.5**
SP_0481	Conserved hypothetical protein		1.3	**1.6**
SP_0491	Hypothetical protein		**0.7**	*0.8*
SP_0492	Conserved domain protein		**0.7**	*0.9*
SP_0494	Ctp synthetase		*1.0*	**3.2**
SP_0503	Hypothetical protein		1.3	**1.9**
SP_0504	Hypothetical protein		1.3	**1.7**
SP_0505	Type I restriction-modification system, S subunit, putative		**0.7**	NA
SP_0508	Type I restriction-modification system, S subunit	*hsdS*	**0.6**	*1.4*
SP_0509	Ecoe type i restriction modification enzyme m subunit.		**0.7**	*0.9*
SP_0526	Response regulator BlpR	*blpR*	*1.0*	**0.7**
SP_0527	Histidine kinase		1.2	**0.7**
SP_0528	Peptide pheromone BlpC	*blpC*	NA	**0.3**
SP_0529	Transport protein BlpB	*blpB*	NA	**0.2**
SP_0530	Conserved hypothetical protein, truncation		*0.8*	**0.2**
SP_0532	Bacteriocin BlpJ	*blpJ*	NA	**0.5**
SP_0535	Hypothetical protein		NA	**0.3**
SP_0539	Bacteriocin BlpM	*blpM*	NA	**0.3**
SP_0540	BlpN protein	*blpN*	NA	**0.3**
SP_0541	Amphipathic pore-forming peptide precursor	*thmA*	*0.8*	**0.3**
SP_0543	Hypothetical protein		*1.1*	**0.2**
SP_0544	Immunity protein BlpX	*blpX*	*0.9*	**0.3**
SP_0545	Hypothetical protein		NA	**0.3**
SP_0546	BlpZ protein, fusion	*blpZ*	*1.1*	**0.4**
SP_0547	Hypothetical protein		NA	**0.3**
SP_0548	Hypothetical protein		*1.0*	**0.7**
SP_0549	Conserved hypothetical protein		*1.2*	**0.7**
SP_0550	YtmQ protein		*1.0*	**0.6**
SP_0552	Hypothetical protein		**0.6**	0.8
SP_0553	Transcription elongation factor nusa	*nusA*	**0.7**	**0.7**
SP_0554	Conserved hypothetical protein		**0.7**	**0.7**
SP_0556	Translation initiation factor IF-2	*infB*	**0.5**	*0.8*
SP_0582	Hypothetical protein		**1.6**	NA
SP_0608	ABC transporter membrane-spanning permease	*glnP*	**0.4**	**1.8**
SP_0615	Beta-lactam resistance factor	*fibA*	**0.7**	*0.8*
SP_0617	Hypothetical protein		**1.5**	**3.4**
SP_0626	Branched-chain amino acid transport system II carrier protein		1.3	**1.6**
SP_0627	Conserved hypothetical protein		**0.7**	*0.9*
SP_0628	HIT family protein		**0.7**	*0.9*
SP_0637	Hypothetical protein		*0.9*	**1.5**
SP_0641	Serine protease, subtilase family		**0.4**	NA
SP_0645	Phosphotransferase system sugar-specific EII component	*PTS-EII*	**0.3**	NA
SP_0647	PTS system, IIC component, putative		**1.7**	**0.6**
SP_0684	Hypothetical protein		**0.4**	0.8
SP_0686	Hypothetical protein.		NA	**0.4**
SP_0687	Abc transporter atp binding subunit.		**0.4**	**0.6**
SP_0690	Cell division protein divib	*divIB*	**0.6**	*0.9*
SP_0704	Hypothetical protein		NA	**1.6**
SP_0715	Hypothetical protein		**0.5**	*1.1*
SP_0717	Hydroxyethylthiazole kinase	*thiM*	**0.6**	NA
SP_0718	Thiamine-phosphate pyrophosphorylase	*thiE*	**0.6**	**0.4**
SP_0726	Phosphomethylpyrimidine kinase		**0.4**	*0.8*
SP_0730	Pyruvate oxidase	*spxB*	**0.4**	0.8
SP_0737	Sodium-dependent transporter	*NS_transporter*	**4.8**	NA
SP_0738	Conserved domain protein		**1.6**	NA
SP_0755	Peptide chain release factor 2 (rf-2).		0.8	**0.7**
SP_0758	Hypothetical protein		**0.5**	0.8
SP_0762	S-adenosylmethionine synthetase	*metK*	**1.5**	1.2
SP_0766	Manganese co-factored superoxide dismutase.		**0.7**	**0.6**
SP_0775	30s ribosomal protein s16.		**2.2**	*1.2*
SP_0776	KH domain protein		**1.6**	*1.1*
SP_0782	Conserved hypothetical protein		1.2	**0.7**
SP_0783	Hypothetical protein		**2.1**	**3.3**
SP_0784	Glutathione reductase		**0.6**	*1.1*
SP_0798	DNA-binding response regulator, CiaR	*ciaR*	*0.9*	**2.2**
SP_0799	Sensor histidine kinase, CiaH	*ciaH*	*1.1*	**2.5**
SP_0800	Hypothetical protein		**2.1**	*1.1*
SP_0803	Rod shape-determining protein roda, putative		1.2	**1.6**
SP_0811	Degenerate transposase	*transposase_C*	**0.7**	NA
SP_0816	Hypothetical protein		**0.7**	*0.9*
SP_0819	Transposase	*transposase_B*	1.2	**0.7**
SP_0820	ATP-dependent Clp protease, ATP-binding subunit ClpE	*clpE*	**0.6**	*0.9*
SP_0822	Conserved hypothetical protein		**1.6**	**1.5**
SP_0824	Glutamine abc transporter, atp-binding protein (GlnQ)	*glnQ*	1.0	**1.5**
SP_0833	Hypothetical protein		**0.7**	*0.9*
SP_0848	ABC transporter membrane-spanning permease	*ABC-MSP*	**1.5**	1.3
SP_0851	Conserved hypothetical protein		**1.5**	*2.2*
SP_0855	Topoisomerase IV, subunit A	*parC*	*1.0*	**1.6**
SP_0856	Branched-chain amino acid aminotransferase	*ilvE*	1.2	**0.6**
SP_0857	Oligopeptide-binding protein, internal deletion, authentic point mutation		1.4	**0.7**

Locus	Common name	Gene Symbol	Ratio index	
			TIGR4	G54
SP_0859	Hypothetical protein.		1.4	**0.7**
SP_0860	Pyrrolidone-carboxylate peptidase		**1.8**	*0.9*
SP_0867	ABC transporter, ATP-binding protein		**0.4**	**0.7**
SP_0868	Conserved hypothetical protein	*yurX*	**0.7**	**0.3**
SP_0869	YurW protein	*yurW*	**0.4**	**0.6**
SP_0870	Hypothetical protein		**0.4**	**0.7**
SP_0871	YurU protein	*yurU*	**0.6**	**0.4**
SP_0875	Transcriptional regulator (DeoR family)		**0.6**	**0.7**
SP_0879	Hypothetical protein		**1.7**	*3.5*
SP_0886	Ecoe type i restriction modification enzyme m subunit.		**0.7**	*1.0*
SP_0887	Type I restriction-modification system, S subunit, putative		**0.7**	*0.8*
SP_0894	X-pro dipeptidyl-peptidase	*pepX*	**1.5**	1.4
SP_0898	Hypothetical protein		**1.5**	*0.9*
SP_0899	Hypothetical protein		**0.7**	**0.7**
SP_0906	Phosphoenolpyruvate-protein phosphotransferase.		**1.6**	*1.0*
SP_0907	Capsular polysaccharide biosynthesis protein, putative		**0.7**	*1.1*
SP_0915	Putative IS1239 transposase		**0.7**	**0.7**
SP_0921	Conserved hypothetical protein		1.3	**0.7**
SP_0923	Cof family protein		**0.7**	*1.0*
SP_0943	Gid protein	*gid*	**0.7**	*1.2*
SP_0953	Acetyltransferase, GNAT family		**1.6**	1.2
SP_0966	Adherence and virulence protein A	*pavA*	*1.0*	**1.5**
SP_0974	Preprotein translocase, secg subunit, putative		**1.6**	NA
SP_0987	Hypothetical protein		**1.6**	*1.1*
SP_0994	Hypothetical protein		1.2	**2.0**
SP_1003	Conserved domain protein		**0.6**	**2.1**
SP_1012	Conserved hypothetical protein		**1.5**	NA
SP_1013	Aspartate-semialdehyde dehydrogenase	*asd*	**1.5**	**0.6**
SP_1014	Dihydrodipicolinate synthase	*dapA*	1.3	**0.7**
SP_1016	Thiophene and furan oxidation protein thdf	*thdF*	**0.7**	**2.6**
SP_1026	Conserved hypothetical protein		**1.5**	*1.0*
SP_1027	Conserved hypothetical protein		**1.5**	**2.0**
SP_1028	Hypothetical protein		1.3	**1.5**
SP_1029	RNA methyltransferase, TrmA family		*0.8*	**1.7**
SP_1033	Iron-compound ABC transporter, ATP-binding protein		*1.2*	**0.7**
SP_1035	Iron-compound ABC transporter, ATP-binding protein		**0.7**	0.8
SP_1040	Cassette chromosome recombinase b		**0.5**	NA
SP_1045	Conserved hypothetical protein		**0.5**	*0.8*
SP_1050	Transcriptional regulator, putative		**0.7**	NA
SP_1051	Hypothetical protein		**0.5**	NA
SP_1052	Phosphoesterase, putative		**0.7**	NA
SP_1058	Hypothetical protein		*1.1*	**0.7**
SP_1069	Hypothetical protein		**0.6**	NA
SP_1088	Tagatose 1,6-diphosphate aldolase		NA	**0.5**
SP_1111	Hypothetical protein		**1.5**	*1.0*
SP_1117	DNA ligase, NAD-dependent	*ligA*	**0.6**	NA
SP_1118	Thermostable pullulanase	*pulI*	1.2	**1.5**
SP_1121	1,4-alpha-glucan branching enzyme	*glgB*	**0.3**	*1.6*
SP_1122	Glucose-1-phosphate adenylyltransferase		**0.6**	**0.6**
SP_1123	Glycogen biosynthesis protein, GlgD	*glgD*	**1.6**	**0.7**
SP_1127	Hypothetical protein		**1.7**	**1.5**
SP_1128	Phosphopyruvate hydratase	*eno*	**0.7**	*1.2*
SP_1129	Integrase-recombinase, phage integrase family		**0.7**	NA
SP_1130	Transcriptional regulator		**0.6**	NA
SP_1144	Conserved hypothetical protein		**0.7**	NA
SP_1145	Hypothetical protein		**0.7**	NA
SP_1157	Voltage-gated chloride channel family protein		1.4	**1.5**
SP_1175	Conserved domain protein		**0.7**	**2.1**
SP_1180	Ribonucleotide-diphosphate reductase beta subunit	*nrdF*	**0.7**	0.8
SP_1191	Tagatose-6-phosphate kinase	*lacC*	**0.7**	NA
SP_1197	Hypothetical protein		**0.2**	*0.7*
SP_1198	Hypothetical protein		**0.2**	*0.9*
SP_1199	Hypothetical protein		**0.3**	*0.7*
SP_1203	Hypothetical protein		**0.7**	*1.0*
SP_1204	Hypothetical protein		**0.7**	*0.9*
SP_1206	Exodeoxyribonuclease VII, small subunit	*xseB*	**0.7**	0.8
SP_1208	Uridine kinase	*udk*	**1.6**	NA
SP_1210	Hypothetical protein		*1.4*	**1.6**
SP_1216	Hypothetical protein		**0.4**	NA
SP_1219	Dna gyrase subunit A		**0.7**	*0.8*
SP_1220	L-lactate dehydrogenase		**1.5**	**1.5**
SP_1222	Type II restriction endonuclease, putative		**0.7**	NA
SP_1225	VicX protein		**0.7**	**0.7**
SP_1226	Histidine kinase		**0.6**	**0.7**
SP_1227	DNA-binding response regulator		**0.7**	0.8
SP_1228	YfhQ protein		**0.6**	**0.6**
SP_1229	Formate--tetrahydrofolate ligase		*1.0*	**0.7**
SP_1232	Hypothetical protein		**0.7**	*1.1*
SP_1234	Transcriptional regulator, biotin repressor family		1.3	**1.5**
SP_1235	Hypothetical protein		**1.5**	1.3
SP_1242	Amino acid ABC transporter, ATP-binding protein		1.4	**2.0**
SP_1249	Gmp reductase (guac).		*1.1*	**0.4**
SP_1252	Hypothetical protein		NA	**1.8**
SP_1253	Hypothetical protein		NA	**2.0**
SP_1255	3-isopropylmalate dehydratase small subunit		NA	**1.7**
SP_1256	Hypothetical protein		**1.5**	**1.7**
SP_1258	2-isopropylmalate synthase, truncation	*leuA-truncation*	NA	**1.5**
SP_1259	Conserved hypothetical protein		NA	**1.6**
SP_1260	Copper homeostasis protein, CutC		1.3	**1.6**
SP_1261	Hypothetical protein		1.4	**1.8**
SP_1267	LicC protein (LicC)	*licC*	**1.9**	**1.6**
SP_1269	Choline kinase		*1.0*	**1.8**
SP_1270	Alcohol dehydrogenase, zinc-containing		1.3	**1.5**
SP_1271	Hypothetical protein		1.4	**1.6**
SP_1274	LicD2		*1.2*	**1.7**
SP_1277	Aspartate carbamoyltransferase		**0.6**	**0.7**
SP_1278	Pyrimidine operon regulatory protein	*pyrR*	**0.6**	*0.9*
SP_1284	LemA protein		**1.5**	**1.5**
SP_1293	50s ribosomal protein l19		**1.7**	1.2
SP_1297	Probable flavodoxin 1		**1.6**	*1.1*

			Ratio index	
**Locus**	**Common name**	**Gene Symbol**	**TIGR4**	**G54**
SP_1306	NADP-specific glutamate dehydrogenase	*gdhA*	**1.6**	*1.2*
SP_1313	IS66 family element, Orf2, interruption		**1.6**	*1.0*
SP_1325	Oxidoreductase, Gfo-Idh-moca family		**0.6**	NA
SP_1339	Hypothetical protein		**1.7**	*1.0*
SP_1343	Protease II (oligopeptidase B)	*ptrB*	**0.5**	NA
SP_1345	Hypothetical protein		**1.6**	NA
SP_1357	ABC transporter, permease/ATP-binding protein		*1.0*	**0.7**
SP_1358	ABC transporter ATP-binding/membrane-spanning protein - unknown substrate	*ABC-N/P*	0.8	**0.6**
SP_1359	Peptide methionine sulfoxide reductase		*0.9*	**0.7**
SP_1360	Homoserine kinase		**0.7**	**0.6**
SP_1361	Homoserine dehydrogenase		0.8	**0.5**
SP_1387	Spermidine-putrescine ABC transporter, permease protein		*1.0*	**1.5**
SP_1388	Spermidine-putrescine transport system permease protein PotB		*0.9*	**1.5**
SP_1415	Glucosamine-6-phosphate isomerase		**0.6**	*0.8*
SP_1417	PspC protein, degenerate		*1.0*	**0.7**
SP_1426	ABC transporter, ATP-binding protein		**0.7**	NA
SP_1429	Hypothetical protein		1.3	**1.7**
SP_1441	IS66 family element, Orf3, degenerate		NA	**0.5**
SP_1453	Hypothetical protein		**0.6**	NA
SP_1454	Hypothetical protein		**0.7**	*0.8*
SP_1460	Probable amino-acid ATP transporter ATP-binding protein YckI		**0.7**	*0.9*
SP_1461	Amino acid ABC transporter, permease protein		**0.6**	0.8
SP_1462	YusI protein		**0.6**	**0.7**
SP_1463	O6-methylguanine-DNA methyltransferase	*ogt*	**0.6**	0.8
SP_1464	Hypothetical protein		**0.6**	**0.6**
SP_1465	Hypothetical protein		**0.6**	*0.9*
SP_1467	Conserved hypothetical protein		*1.0*	**2.1**
SP_1468	Pyridoxine biosynthesis protein		*1.4*	**2.1**
SP_1470	Thiamine biosynthesis protein apbe, putative		**0.5**	0.8
SP_1471	Hypothetical protein		**0.4**	**0.7**
SP_1472	Oxidoreductase, putative		**0.7**	**0.6**
SP_1478	YvgN protein		*0.9*	**0.7**
SP_1502	Amino acid ABC transporter, permease protein		**1.5**	*1.2*
SP_1510	ATP synthase F1, alpha subunit	*atpA*	**0.7**	1.4
SP_1518	YrrI protein		**0.7**	*1.0*
SP_1523	Orf1 and snf2 gene		*1.0*	**0.7**
SP_1531	Hypothetical protein		*1.2*	**0.6**
SP_1546	Hypothetical protein		**0.6**	**0.7**
SP_1548	Hypothetical protein		*1.0*	**0.7**
SP_1549	Polypeptide deformylase		*0.9*	**0.7**
SP_1550	Glutathione S-transferase family protein		*1.2*	**0.4**
SP_1551	P-type atpase - calcium transporter	*pacL*	*1.0*	**0.4**
SP_1559	YbbT protein		**0.7**	*1.0*
SP_1572	Surface located protein		*1.0*	**1.7**
SP_1576	Homoserine o-succinyltransferase		*1.0*	**0.7**
SP_1578	Hypothetical protein		**0.6**	**0.5**
SP_1580	Multiple sugar-binding transport atp-binding protein, MsmK		**0.4**	*0.9*
SP_1583	YueJ protein		*1.3*	**0.6**
SP_1584	Transcriptional regulator, putative		*1.0*	**0.6**
SP_1586	Autoaggregation-mediating protein		*1.0*	**0.6**
SP_1587	Oxalate-formate antiporter (oxlt-2)		*0.9*	**0.3**
SP_1590	Cobyric acid synthase CobQ		**0.7**	*0.9*
SP_1597	Hypothetical protein		**1.5**	*1.4*
SP_1598	Phosphomethylpyrimidine kinase, putative		**1.9**	1.4
SP_1599	TrnA pseudouridine synthase A		**1.5**	1.3
SP_1601	Conserved hypothetical protein		**1.6**	*1.2*
SP_1602	Required for expression of the phosphonate utilization phenotype in *E. coli*	*phnA*	**1.7**	*1.1*
SP_1623	Cation-transporting ATPase		**1.6**	1.4
SP_1626	Ribosomal protein S15		**2.1**	NA
SP_1633	Response regulator		*0.9*	**1.5**
SP_1647	Endopeptidase O	*pepO*	*0.9*	**1.6**
SP_1650	Manganese ABC transporter, manganese-binding adhesion liprotein	*psaA*	**0.6**	*1.0*
SP_1651	Thiol peroxidase	*psaD*	**0.5**	**0.7**
SP_1675	Xylose operon regulatory protein (xylR-2)		1.4	**0.7**
SP_1676	Putative acylneuraminate lyase		*0.9*	**0.6**
SP_1679	Hypothetical protein		1.2	**0.5**
SP_1681	Sugar abc transporter, permease protein.		**1.9**	**0.6**
SP_1682	Sugar abc transporter, permease protein.		*1.2*	**0.5**
SP_1683	Hypothetical protein		**0.5**	**0.4**
SP_1684	PTS system, glucose-specific IIBC component	*ptsG*	**0.3**	**0.6**
SP_1685	N-acetylmannosamine-6-phosphate 2-epimerase	*nanE*	**0.2**	*1.0*
SP_1691	Conserved hypothetical protein		**0.2**	NA
SP_1693	Sialidase a precursor		**0.4**	NA
SP_1694	Hypothetical protein		**0.5**	*1.2*
SP_1695	Xylan esterase 1		NA	**3.9**
SP_1696	Hypothetical protein		1.3	**2.6**
SP_1710	Homologue of NADPH-flavin oxidoreductase	*frp*	**0.7**	0.8
SP_1711	Primosomal protein DnaI		0.8	**0.7**
SP_1712	Hypothetical protein		**0.7**	**0.7**
SP_1713	Conserved hypothetical protein		**0.7**	**0.7**
SP_1715	ABC transporter, ATP-binding protein		**0.6**	*1.1*
SP_1719	Transposase	*IS1239*	**0.6**	**0.6**
SP_1753	Hypothetical symporter hi1545		**1.5**	*0.8*
SP_1754	Conserved hypothetical protein		1.3	**0.7**
SP_1764	Glycosyl transferase, family 2		**1.5**	NA
SP_1769	Glycosyl transferase, authentic frameshift		**1.7**	NA
SP_1771	Glycosyl transferase, family 2-glycosyl transferase family 8		**1.5**	NA
SP_1774	Transcriptional regulator, putative		**0.7**	**0.7**
SP_1775	Hypothetical protein		**0.6**	**0.7**
SP_1776	Thioredoxin reductase	*trxA*	**0.6**	**0.6**
SP_1778	Water channel protein		*1.0*	**0.6**
SP_1782	Ribosomal protein L11 methlytransferase		**0.7**	*1.3*
SP_1790	ATPase, AAA family	*yrvN*	*1.1*	**1.5**
SP_1793	Hypothetical protein		**1.6**	NA

Locus	Common name	Gene Symbol	Ratio index TIGR4	Ratio index G54
SP_1801	Hypothetical protein		**0.5**	*0.9*
SP_1802	Hypothetical protein		**0.4**	**0.7**
SP_1803	Conserved hypothetical protein		**0.4**	**0.6**
SP_1804	General stress protein 24, putative		**0.4**	**0.7**
SP_1815	Anthranilate phosphoribosyltransferase		*0.7*	**0.7**
SP_1837	Putative capsular polysaccharide biosynthesis protein		*0.9*	**1.5**
SP_1839	ABC transporter ATP-binding/membrane spanning protein - unknown substrate		*1.0*	**1.5**
SP_1845	Exodeoxyribonuclease	*exoA*	**0.7**	**0.6**
SP_1846	Conserved hypothetical protein, degenerate		**0.7**	**0.7**
SP_1849	DpnD protein	*dpnD*	**1.6**	NA
SP_1853	Galactokinase	*galK*	*0.9*	**0.5**
SP_1855	Dehydrogenase		*1.0*	**0.4**
SP_1856	Transcriptional regulator, MerR family		*1.0*	**0.4**
SP_1857	Cation efflux system protein		*1.0*	**0.6**
SP_1858	Hypothetical protein		**1.5**	NA
SP_1860	Choline transporter	*proWX*	**0.5**	0.8
SP_1861	ABC transporter ATP-binding protein - choline transporter	*proV*	**0.5**	0.8
SP_1862	Hypothetical protein		**0.5**	**0.6**
SP_1872	Ferric anguibactin-binding protein precusor FatB of *V. anguillarum*		*0.9*	**0.4**
SP_1888	Oligopeptide ABC transporter, ATP-binding protein AmiE	*amiE*	*1.0*	**0.7**
SP_1889	Oligopeptide ABC transporter, permease protein AmiD	*amiD*	*1.1*	**0.6**
SP_1890	Oligopeptide transport system permease protein AmiC		*1.1*	**0.7**
SP_1891	ABC transporter substrate-binding protein - oligopeptide transport	*amiA*	*1.1*	**0.6**
SP_1893	Hypothetical protein		**1.5**	**1.5**
SP_1905	IS1167, transposase, interruption		**1.5**	1.2
SP_1906	Chaperonin, 60 kda	*groEL*	0.8	**0.7**
SP_1907	Cochaperonin groes		**0.7**	*0.9*
SP_1922	Conserved hypothetical protein		**1.5**	*0.9*
SP_1923	Pneumolysin	*ply*	*0.9*	**0.4**
SP_1924	Hypothetical protein		*0.7*	**0.4**
SP_1925	Hypothetical protein		**0.6**	**0.4**
SP_1926	Hypothetical protein		**0.6**	**0.5**
SP_1937	Autolysin	*lytA*	*1.1*	**0.7**
SP_1940	RecA protein		0.8	**0.6**
SP_1941	Competence-damage inducible protein, putative		*0.8*	**0.6**
SP_1942	Membrane-bound protein LytR		*1.2*	**0.7**
SP_1943	Hypothetical protein		0.8	**0.7**
SP_1946	Hypothetical protein		*1.2*	**0.6**
SP_1952	Hypothetical protein		**0.5**	NA
SP_1954	Serine protease, subtilase family, authentic frameshift		**0.5**	NA
SP_1956	Hypothetical protein		**0.7**	*0.8*
SP_1957	Abc transporter atp binding subunit.		**0.7**	0.8
SP_1972	Membrane protein		*1.1*	**1.5**
SP_1975	Oxaa-like protein precursor		1.4	**1.7**
SP_1976	Pyruvate formate-lyase-activating enzyme		**0.7**	*0.8*
SP_1979	Pur operon repressor	*purR*	**0.7**	*0.9*
SP_1990	Hypothetical protein		**0.7**	*1.1*
SP_1991	Putative hydrolase		**0.6**	*1.1*
SP_1993	Ribosomal protein L34	*rpmH*	**1.6**	*1.3*
SP_2026	Alcohol-acetaldehyde dehydrogenase		**0.6**	**0.3**
SP_2041	Stage iii sporulation protein J precursor.		*1.1*	**0.7**
SP_2054	Hypothetical protein		**0.5**	*0.8*
SP_2055	Alcohol dehydrogenase		**0.2**	**0.6**
SP_2058	Queuine trna-ribosyltransferase	*tgt*	1.3	**2.5**
SP_2063	Lysm domain protein		1.4	**1.6**
SP_2069	Glutamyl-tRNA synthetase		**0.7**	*0.9*
SP_2070	Glucose-6-phosphate isomerase	*pgi*	*1.1*	**1.6**
SP_2092	UTP-glucose-1-phosphate uridylyltransferase		**0.7**	NA
SP_2093	Hypothetical protein		**0.7**	NA
SP_2094	Conserved hypothetical protein		**1.5**	1.3
SP_2101	Cation-transporting atpase, EI-E2 family		**0.7**	**0.7**
SP_2108	Maltose-maltodextrin-binding protein precursor		**0.4**	*0.8*
SP_2109	Maltodextrin transport system permease protein MalC	*malC*	**0.6**	**0.6**
SP_2110	Maltodextrin ABC transporter, permease protein	*malD*	*1.3*	**0.7**
SP_2116	Hypothetical protein		**1.5**	*0.9*
SP_2119	Hypothetical protein		NA	**0.6**
SP_2125	Conserved hypothetical protein		**1.5**	**0.7**
SP_2131	Transcriptional regulator, BglG family		**0.4**	NA
SP_2132	Hypothetical protein		**0.6**	**0.6**
SP_2136	PcpA protein	*pcpA*	**0.5**	**1.5**
SP_2143	Hypothetical protein		NA	**0.7**
SP_2157	Alcohol dehydrogenase		NA	**0.6**
SP_2163	Phosphotransferase system sugar-specific EII component	*PTS-EII*	**0.4**	NA
SP_2167	Fucose kinase	*fcsK*	0.9	**0.5**
SP_2170	Zinc ABC transporter, permease protein	*adcB*	*1.0*	**1.9**
SP_2171	Zinc ABC transporter, ATP-binding protein	*adcC*	*1.1*	**1.7**
SP_2172	Adc operon repressor, adcR	*adcR*	*1.2*	**1.5**
SP_2173	Extramembranal protein		1.3	**2.3**
SP_2174	D-alanyl carrier protein	*dltC*	1.3	**2.5**
SP_2175	Integral membrane protein.		*1.0*	**2.6**
SP_2176	D-alanine-activating enzyme	*dltA*	*1.0*	**2.3**
SP_2177	Hypothetical protein		1.4	**2.3**
SP_2184	Glycerol uptake facilitator protein		NA	**0.7**
SP_2187	Hypothetical protein		**0.4**	*1.1*
SP_2189	TIM-barrel protein, putative, nifr3 family		**0.6**	*1.0*
SP_2190	SpsA protein		**0.6**	NA
SP_2196	Abc transporter, ATP-binding protein.		**0.6**	**0.7**
SP_2197	ABC transporter, substrate-binding protein, putative		**0.5**	**0.6**
SP_2198	ABC transporter, permease protein		**0.5**	NA
SP_2199	Hypothetical protein		**0.7**	**0.7**
SP_2206	Ribosomal subunit interface protein	*yfiA*	**0.5**	1.4
SP_2236	Putative sensor histidine kinase comd	*comD*	**2.0**	*1.0*
SP_2237	Competence stimulating peptide precursor (CSP)	*comC*	**1.5**	*1.0*
SP_2239	HtrA protein	*htrA*	*0.8*	**1.9**
