# Supplementary material for: Phenotypic, genomic, and transcriptional characterization of Streptococcus pneumoniae interacting with human pharyngeal cells
Source: BMC Genomics. 2013 Jun 9;14:383. doi: 10.1186/1471-2164-14-383 (PMC3708772; doi:10.1186/1471-2164-14-383)

Additional data file 9. Comparison of RNA-Seq average coverage (X axis) to qRT-PCR cycle thresholds (Ct, Y axis) for a subset of 21 pneumococcal genes. The colors respresent three independent replicates of qRT-PCR.

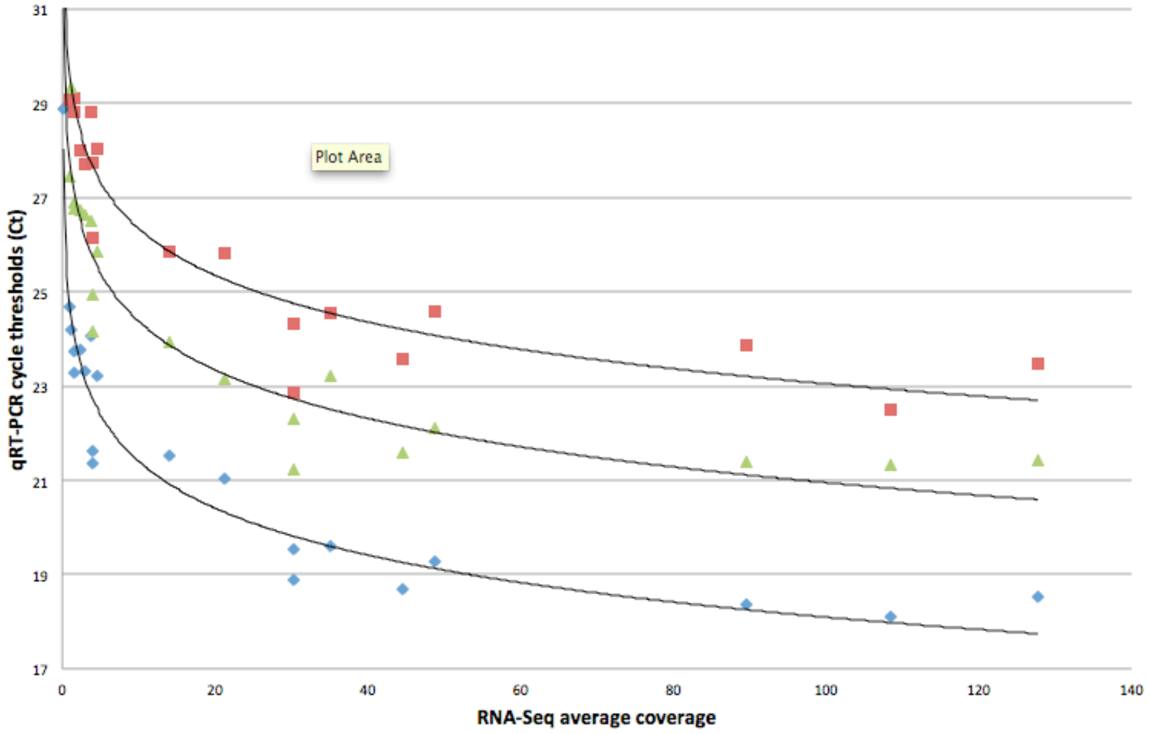

Supplement: Additional file 9 — Is a figure showing the correlation between RNA-Seq average coverage and qRT-PCR thresholds. [file 1471-2164-14-383-S9.pdf]
