# Supplementary material for: Phenotypic, genomic, and transcriptional characterization of Streptococcus pneumoniae interacting with human pharyngeal cells
Source: BMC Genomics. 2013 Jun 9;14:383. doi: 10.1186/1471-2164-14-383 (PMC3708772; doi:10.1186/1471-2164-14-383)
Supplement: Additional file 11 — Is a table showing the hemolytic activity of pneumolysin in TIGR4 wild type and its SP_1922 isogenic mutant strain. [file 1471-2164-14-383-S11.pdf]

Additional data file 11. Hemolytic activity of pneumolysin in TIGR4 wild type and its SP_1922 isogenic mutant strain. Human erythrocytes were exposed to various conditions and percent lysis was determined using a control containing 2% (v/v) erythrocytes in de-ionized water.

Condition	10-fold serial dilutions of bacterial cell extracts	% Lysis
TIGR4 wild type	10%	100
	1%	63
	0.10%	10
TIGR4_1922_knockout	10%	100
	1%	75
	0.10%	9
**Condition**	**Concentration**	**% Lysis**
Saponin (Positive control)	1%	100
	0.10%	100
	0.01%	100
	0.001%	73
	0.0001%	11
DMSO (Negative control)	1%	11
	0.10%	13
	0.01%	13
	0.001%	10
	0.0001%	19
dH_20 (Positive control)	N/A	100
